# Supplementary material for: Exercise‐induced α‐ketoglutaric acid stimulates muscle hypertrophy and fat loss through OXGR1‐dependent adrenal activation
Source: EMBO J. 2020 Feb 27;39(7):e103304. doi: 10.15252/embj.2019103304 (PMC7110140; doi:10.15252/embj.2019103304)
Supplement: Supplementary file 1 — Appendix [file EMBJ-39-e103304-s001.pdf]

α-ketoglutaric acid stimulates muscle hypertrophy and fat loss through OXGR1-dependent adrenal activation

Yexian Yuan[1, 9], Pingwen Xu[3, 9], Qingyan Jiang[1, 2, 9], Xingcai Cai[1], Tao Wang[1], Wentong Peng[1], Jiajie Sun[1], Canjun Zhu[1], Cha Zhang[1], Dong Yue[1], Zhihui He[1], Jinping Yang[1], Yuxian Zeng[1], Man Du[1], Fenglin Zhang[1], Lucas Ibrahimi[3], Sarah Schaul[3], Yuwei Jiang[4], Jiqiu Wang[5], Jia Sun[6], Qiaoping Wang[7], Liming Liu[8], Songbo Wang[1], Lina Wang[1], Xiaotong Zhu[1], Ping Gao[1], Qianyun Xi[1], Cong Yin[1], Fan Li[1], Guli Xu[1], Yongliang Zhang[1], Gang Shu[1, 2, *,†]

Appendix

Contents

Appendix Figure S1: The metabolic phenotype of OXGR1 KO mouse on normal chow………3

Appendix Figure S2. Adrenal specific reexpression of OXGR1 rescues the stimulatory effects of AKG on thermogenesis and lipolysis…………………………………………………………..5

Appendix Figure S3. Adrenal specific overexpression of OXGR1 enhances stimulatory effects of AKG on thermogenesis and lipolysis…………………………….……………………………..7

Appendix Table S1. Clinical characteristics of all human subjects……………………........9

Appendix Table S2. PCR primer sequences of related genes……………………………….10

Appendix Figure S1

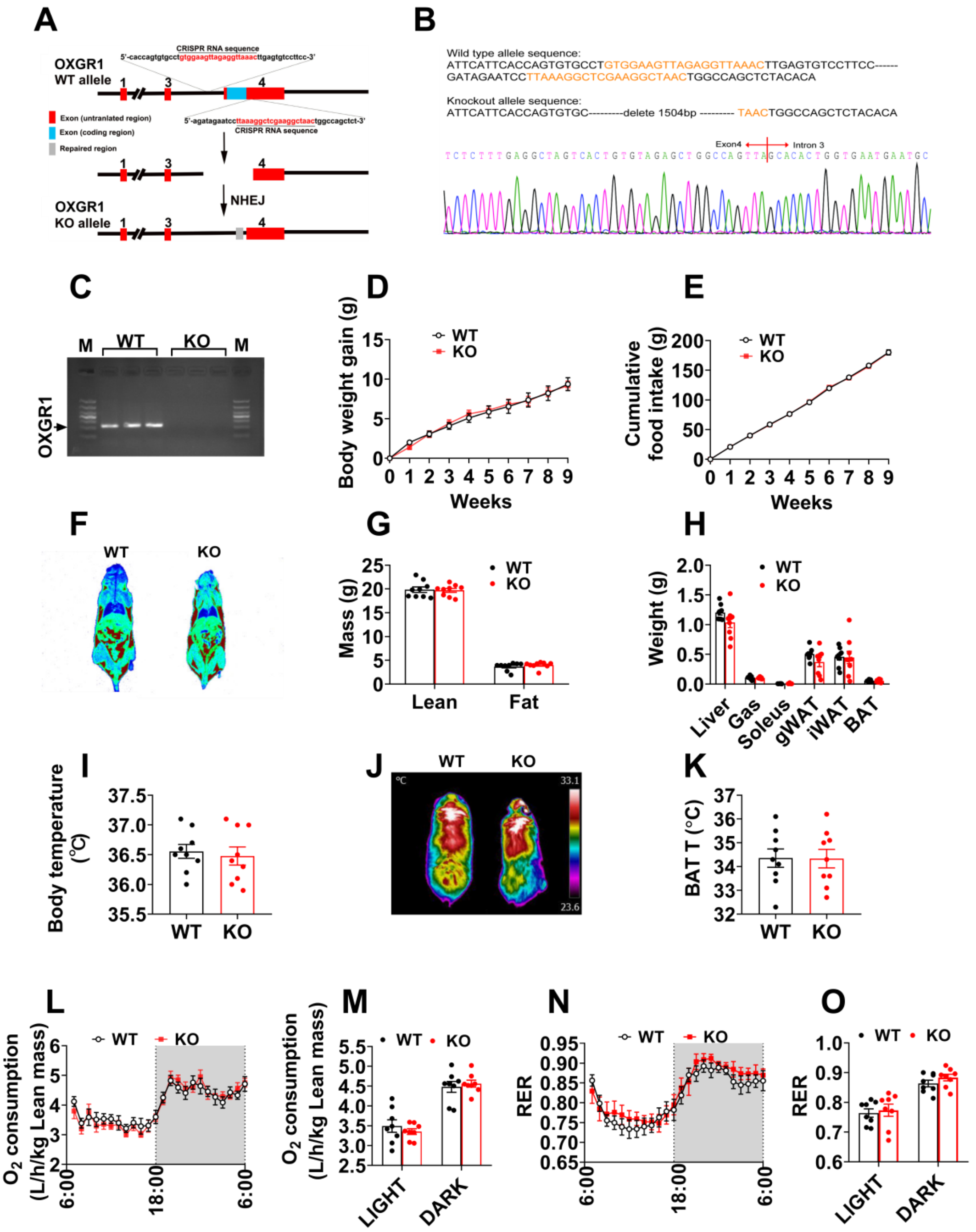

Appendix Figure S1. The metabolic phenotype of OXGR1 KO mouse on normal chow

(A). Schematic representation of OXGR1KO mouse line generation by Clustered Regularly Interspaced Short Palindromic Repeats (CRISPR) strategy. The sgRNA sites were located in intron 3 and exon 4 of the OXGR1 gene. The DNA sequences contained sgRNA-binding regions are labeled with red.

(B). The genomic sequencing of sgRNA target sites in wild-type and OXGR1KO mice. The orange letter is the sgRNA target sequence.

(C). The validation of OXGR1KO mice. The mRNA expression of OXGR1 was determined in the adrenal glands from male WT control (littermates) or OXGR1KO mice.

(D-E). Body weight gain (D) and cumulative food intake (E) of male OXGR1 KO mice and littlermates. Chow fed male mice (8 weeks of age) were monitored for 9 weeks (n = 8 per group).

(F-H). Representative images (F) of body composition and fat and lean mass (G) and tissue weight (H) of male OXGR1KO mice after 9-weeks of monitoring (n = 8 per group).

(I). The body temperature of male OXGR1KO mice after 9-weeks of monitoring (n = 8 per group).

(J-K). Representative images (J) and quantification (K) of BAT thermogenesis induced by 6 hrs cold exposure at 4°C in male OXGR1KO mice after 9-weeks of monitoring (n = 8 per group).

(L-O). Oxygen consumption (L-M) and RER (N-O) in male OXGR1KO mice after 9-weeks of monitoring (n = 8 per group).

Data information: Results are presented as mean ± SEM. In (D-E), (L) and (N) data was analyzed by two-way ANOVA followed by post hoc Bonferroni tests. In (G-I), (K), (M) and (O), data was analyzed by non-paired Student's t-test.

Appendix Figure S2

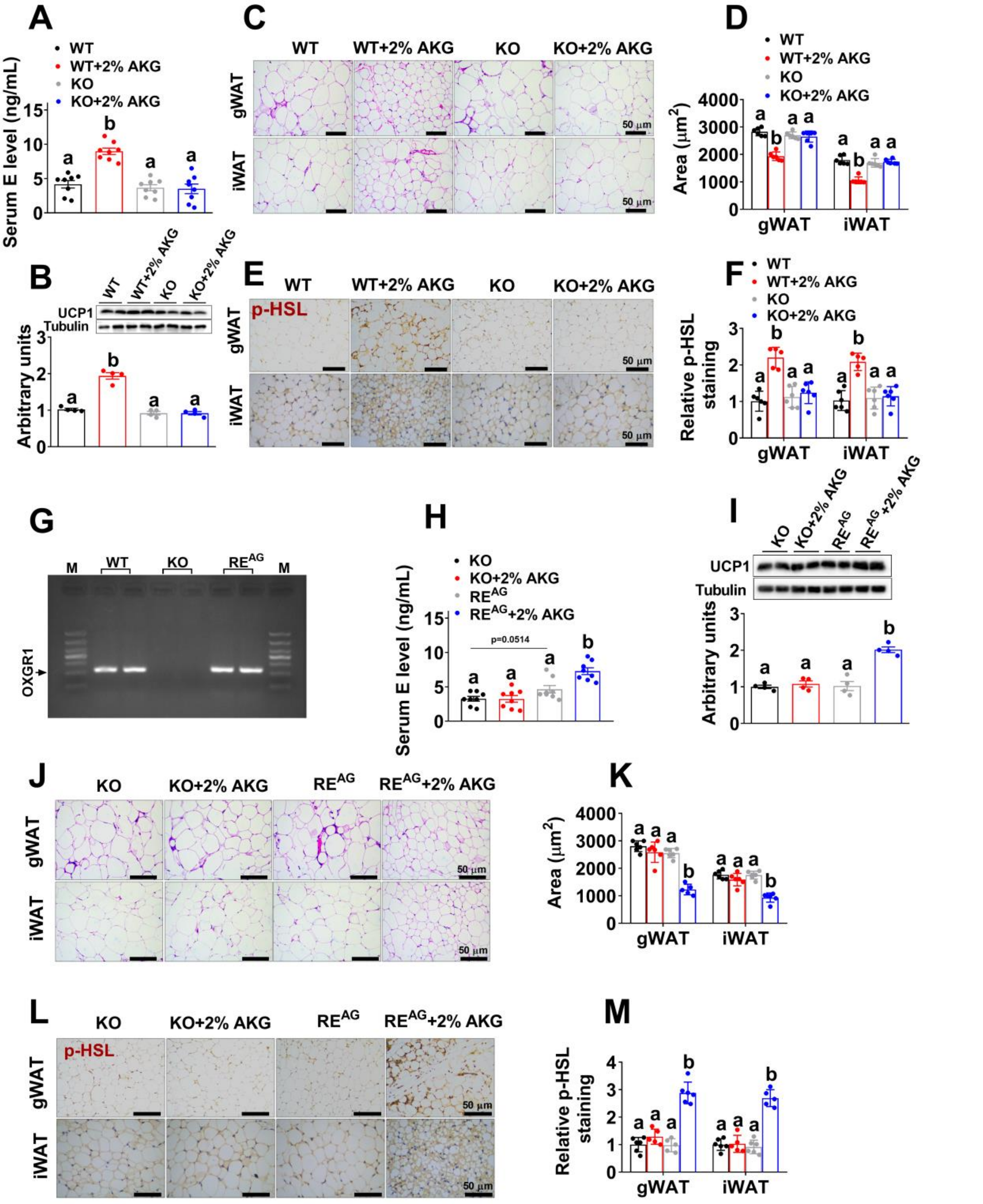

Appendix Figure S2. Adrenal specific reexpression of OXGR1 rescues the stimulatory effects of AKG on thermogenesis and lipolysis

(A). Serum E level in male OXGR1KO mice. At 12 weeks of age, male control or OXGR1KO mice were switched to HFD and received tap water or water supplemented with 2% AKG for 13 weeks (n = 8 per group).

(B). Immunoblots and quantification of UCP1 protein expression in the BAT of male OXGR1KO mice treated with AKG for 13 weeks (n = 4 per group).

(C-D). Representative images (C) and quantification (D) of iWAT and gWAT HE staining from male OXGR1KO mice treated with AKG for 13 weeks (n = 6 per group).

(E-F). Representative images (E) and quantification (F) of p-HSL DAB staining from male OXGR1KO mice treated with AKG for 13 weeks (n = 6 per group).

(G). The validation of OXGR1 reexpression. The mRNA expression of OXGR1 was determined in the adrenal glands from male WT control, OXGR1KO injected with HBAAV2/9-GFP, and OXGR1KO injected with HBAAV2/9-OXGR1 ($OXGR1RE^{AG}$) mice.

(H). Serum E level in male $OXGR1RE^{AG}$. Male OXGR1KO mice (8 weeks) were adrenal-specifically injected with control HBAAV2/9-GFP or HBAAV2/9-OXGR1. Two weeks after injections, mice were switched to HFD and further divided into two groups, receiving tap water or 2% AKG for 13 weeks. (n = 6 per group).

(I). Immunoblots and quantification of UCP1 protein expression in the BAT of $OXGR1RE^{AG}$ mice treated with AKG for 13 weeks (n = 4 per group).

(J-K). Representative images (J) and quantification (K) of iWAT and gWAT HE staining from $OXGR1RE^{AG}$ mice treated with AKG for 13 weeks (n = 6 per group).

(L-M). Representative images (L) and quantification (M) of p-HSL DAB staining from $OXGR1RE^{AG}$ mice treated with AKG for 13 weeks (n = 6 per group).

Data information: Results are presented as mean ± SEM. In (A-B), (D), (F), (H-I), (K) and (M), different letters between bars indicate $p \leq 0.05$ by one-way ANOVA followed by post hoc Turkey's tests.

Appendix Figure S3

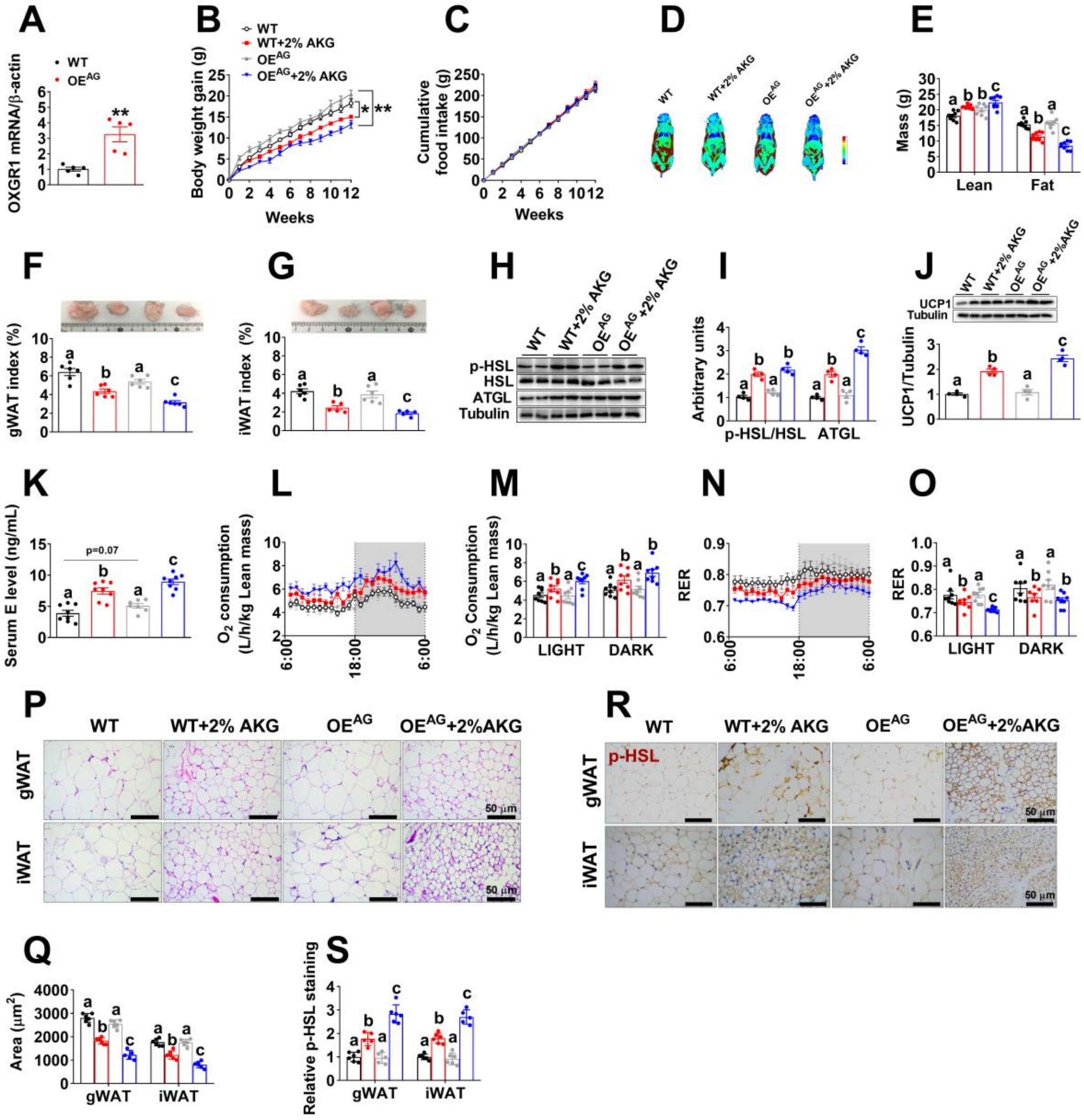

Appendix Figure S3. Adrenal specific overexpression of OXGR1 enhances stimulatory effects of AKG on thermogenesis and lipolysis

(A). The validation of OXGR1 overexpression. The mRNA expression of OXGR1 was determined in the adrenal glands from male WT control, WT injected with HBAAV2/9-GFP, and WT injected with HBAAV2/9-OXGR1 (OXGR1OE^{AG}) mice (n=5 per group).

(B-C). Body weight gain (B) and cumulative food intake (C) of OXGR1OE^{AG}. Male C57BL/6 mice (8 weeks) were adrenal-specifically injected with control HBAAV2/9-GFP or HBAAV2/9-OXGR1. Two weeks after injections, mice were switched to HFD and further divided into two groups, receiving tap water or water supplemented with 2% AKG for 12 weeks (n = 8 per group).

(D-E). Representative image of body composition (D) and fat and lean mass index (E) of male OXGR1OE^{AG} mice treated with AKG for 12 weeks (n = 8 per group).

(F-G). Weight index of gWAT (F) and iWAT (G) in male OXGR1OE^{AG} mice treated with AKG for 12 weeks (n = 6 per group).

(H-I). Immunoblots (H) and quantification (I) of p-HSL and ATGL protein in the gWAT of male OXGR1OE^{AG} mice treated with AKG for 12 weeks (n = 4 per group).
(J). Immunoblots and quantification of UCP1 protein in the BAT of male OXGR1OE^{AG} mice treated with AKG for 12 weeks (n = 4 per group).
(K). Serum E level in male OXGR1OE^{AG} mice treated with AKG for 12 weeks (n= 8 per group).
(L-O). Oxygen consumption (L-M) and RER (N-O) of male OXGR1OE^{AG} mice treated with AKG for 12 weeks (n = 8 per group).
(P-Q). Representative images (P) and quantification (Q) of gWAT and iWAT HE staining from male OXGR1OE^{AG} mice treated with AKG for 12 weeks (n = 6 per group).
(R-S). Representative images (R) and quantification (S) of p-HSL DAB staining from male OXGR1OE^{AG} mice treated with AKG for 12 weeks (n = 6 per group).
Data information: Results are presented as mean ± SEM. In (A), ** $p \leq 0.01$ by non-paired Student's t test. In (B-C), *$p \leq 0.05$, **$p \leq 0.01$ by two-way ANOVA followed by post hoc Bonferroni tests. In (E-G), (I-K), (M), (O), (Q) and (S), different letters between bars indicate $p \leq 0.05$ by one-way ANOVA followed by post hoc Turkey's tests.

Appendix Table S1. Clinical characteristics of all human subjects.

NO.	Gender	Age	Height (cm)	Weight (kg)	BMI (kg/m^2)	Fat mass (%)	VFA (cm^2)	NCF (cm)	WCF (cm)	HCF (cm)	Blood pressure (mmHg)
1	F	48	154	61.7	26.02	37.7	111.8	33.9	85.9	99.3	113.5/91
2	F	44	159.5	58.2	22.88	31.6	80.6	33.4	78.5	87.4	106/76
3	F	47	160.5	63.4	24.61	35.3	98.6	36	80	95	114/82
4	F	42	165.5	64.7	23.62	35.3	96.1	33.5	83.5	98.5	113.5/88
5	F	40	176.5	75.3	24.17	38.9	101.4	33	83.5	104.5	113/78
6	F	42	161	66.2	25.54	38.9	105.2	32.5	87	100.9	117/74
7	F	36	168.5	89.8	31.63	41.5	140.5	37	96	113.5	159/107
8	F	32	157.8	66	26.51	37	89.1	35.5	79	100	95/67
9	F	35	168	71.5	25.33	32.1	87.8	36.5	84.5	102	118/87
10	F	36	164.7	75.5	27.83	40.4	110.7	34	84.5	109	112/74
11	F	32	163	65.3	24.58	30.1	74.8	33.5	85	99.5	132/90.5
12	F	28	160	66.2	25.86	30.7	72.3	34	86.5	97	109/71
13	F	31	155.5	63.6	26.3	40.1	97.9	33.9	94	100	101/69
14	F	29	170.5	71.6	24.63	42.1	102.2	34	80	105.5	105/75
15	F	30	161.5	64	24.54	34.4	74.8	32.8	82.5	94	106/75
16	F	31	161.3	71.9	27.64	39.4	100	34	92	106	/
17	F	30	162.5	72.1	27.3	36.8	98.9	36.5	95	103.5	112/69
18	F	27	164.5	70.1	25.91	36.5	91.8	33	86	105	126/82
19	F	31	159	63.5	25.12	36.8	76.1	33.8	79.5	97.9	/
20	F	29	174.8	87.5	28.64	38.4	126.2	38	99.5	112.5	/
21	F	30	162.5	76.3	28.89	41.5	108.6	36	93.9	105	110/78
22	F	27	161.7	61.8	23.64	36.9	78.2	33	76.2	96	123/82
23	F	51	162	62.2	23.7	34.7	98.4	34	81.5	96.5	117/79
24	F	49	160.5	63.6	24.69	34	96.2	33	83.5	98.2	130/82
25	F	43	161.5	61.4	23.54	35.2	100.1	34	78.5	96	104/78
26	F	49	163.5	69.9	26.15	38.1	115.3	33.9	86	103.5	115/78
27	F	29	160	68.8	26.88	42.7	98	34.5	88	102.9	108/65
28	F	46	162.2	69.5	26.42	43.4	126.8	35.5	92.5	104.5	120/80
29	F	29	169.5	80.1	27.88	36.2	106.5	34.9	93.5	106	114/66
30	F	38	158	81.5	32.65	40.6	144.7	37	99.5	110.5	124/87
31	F	46	167.8	77.2	27.42	35.5	123.6	37.5	91	106.5	116/77
32	F	47	156.2	59.3	24.3	33.2	90.5	31	72.5	98.7	88/61
33	F	43	156.3	70.8	28.98	41.4	126.1	37	87.5	100	123/88
34	M	43	170	78	27	/	/	/	/	/	/
35	F	57	150	50	22	/	/	/	/	/	/
36	M	61	168	58	20.5	/	/	/	/	/	/
37	M	44	163	62	23.3	/	/	/	/	/	/
38	M	52	145	49.5	23.5	/	/	/	/	/	/
39	M	24	160	61	23.8	/	/	/	/	/	/
40	M	45	160	65	25.3	/	/	/	/	/	/
41	M	71	161	81	31.2	/	/	/	/	/	/
42	M	57	174	77.5	25.6	/	/	/	/	/	/
43	M	76	167	65	23.3	/	/	/	/	/	/
44	M	37	151	51.3	22.5	/	/	/	/	/	/
45	F	82	157	60	24.3	/	/	/	/	/	/

BMI: body mass index as the body mass divided by the square of the body height; VFA: visceral fat area; NCF: neck circumference; WCF: waist circumference; HCF: hip circumference.

Appendix Table S2. PCR primer sequences of related genes

Gene abbreviation	Forward Primer （5’-3’）	Reverse Primer （5’-3’）
UCP1	ACTGCCACACCTCCAGTCATT	CTTTGCCTCACTCAGGATTGG
Cidea	TGCTCTTCTGTATCGCCCAGT	GCCGTGTTAAGGAATCTGCTG
Dio2	AATTATGCCTCGGAGAAGACCG	GGCAGTTGCCTAGTGAAAGGT
ATGL	ACACCAGCATCCAGTTCAACCTTC	GACATCAGGCAGCCACTCCAAC
HSL	CTCCTCATGGCTCAACTCC	ACTCCTGCGCATAGACTCC
PPARγ	GGAAGACCACTCGCATTCCTT	GTAATCAGCAACCATTGGGTCA
FASN	CTCCAAGCAGGCGAACACG	CGAAGGGAAGCAGGGTTGAT
ACC	TGATTCTCAGTTCGGGCACT	CTCTGCCTGCACTTTCTCTG
CD137	CACGGAGCTCATCTCTTGGT	GTCCACCTATGCTGGAGAAGG
TBX1	TGGGACGAGTTCAATCAGCT	CACAAAGTCCATCAGCAGCA
TMEM26	ACCCTGTCATCCCACAGAG	TGTTTGGTGGAGTCCTAAGGTC
slc27a1	CGCTTTCTGCGTATCGTCTG	GATGCACGGGATCGTGTCT
CD40	TTGTTGACAGCGGTCCATCTA	CCATCGTGGAGGTACTGTTTG
CITED1	GAGGCCTGCACTTGATGTC	CACGGAGCTCATCTCTTGGT
JMJD3	CACCCCAGCAAACCATATTATGC	CACACAGCCATGCAGGGATT
OXGR1	CTGCCTGCCATTGGTGATAGTGAC	TGCCTGCTGGAAGTTATTGCTGAC
PHD1	AAGTGGTATGGGCTGTGACA	GTCAACATGCCTCACGTACC

PHD2	AGGCAACGGAACAGGCTATG	CGCATCTTCCATCTCCATTTG
PHD3	ACCTGCAGACGACATCCTAG	TGTGAGGGTTTGGAGGGTAC
OCT4	GAGGAGTCCCAGGACATGAA	AGATGGTGGTCTGGCTGAAC
ASZ1	AAGTGCTTGTTCTGCTCGTG	CATCCTGGGCGTTAACTTCG
Wfdc15a	TGAAGCCAAGCAGCCTCCTA	AGGTTGTCCAGGGTTCCACA
TET1	AGCTACCCTGAGTTTCACCC	CAATTAGGCGCTGTCTGTCC
TET2	TGTGTGGCACTAGATTTCAT	AGTCTCTGAAGCCTGTTGAT
TET3	CAGTGGCTTCTTGGAGTCACCTC	GGATGGCTTTCCCCTTCTCTCC
Dazl	TGCAGCCTCCAACCATGATGAATC	CACTGTCTGTATGCTTCGGTCCAC
UTX	AAGGCTGTTCGCTGCTACG	GGATCGACATAAAGCACCTCC
β-actin	CCACTGGCATCGTGATGGACTCC	GCCGTGGTGGTGAAGCTGTAGC
