# Supplementary material for: Exercise‐induced α‐ketoglutaric acid stimulates muscle hypertrophy and fat loss through OXGR1‐dependent adrenal activation
Source: EMBO J. 2020 Feb 27;39(7):e103304. doi: 10.15252/embj.2019103304 (PMC7110140; doi:10.15252/embj.2019103304)
Supplement: Supplementary file 2 — Expanded View Figures PDF [file EMBJ-39-e103304-s002.pdf]

Expanded View Figures

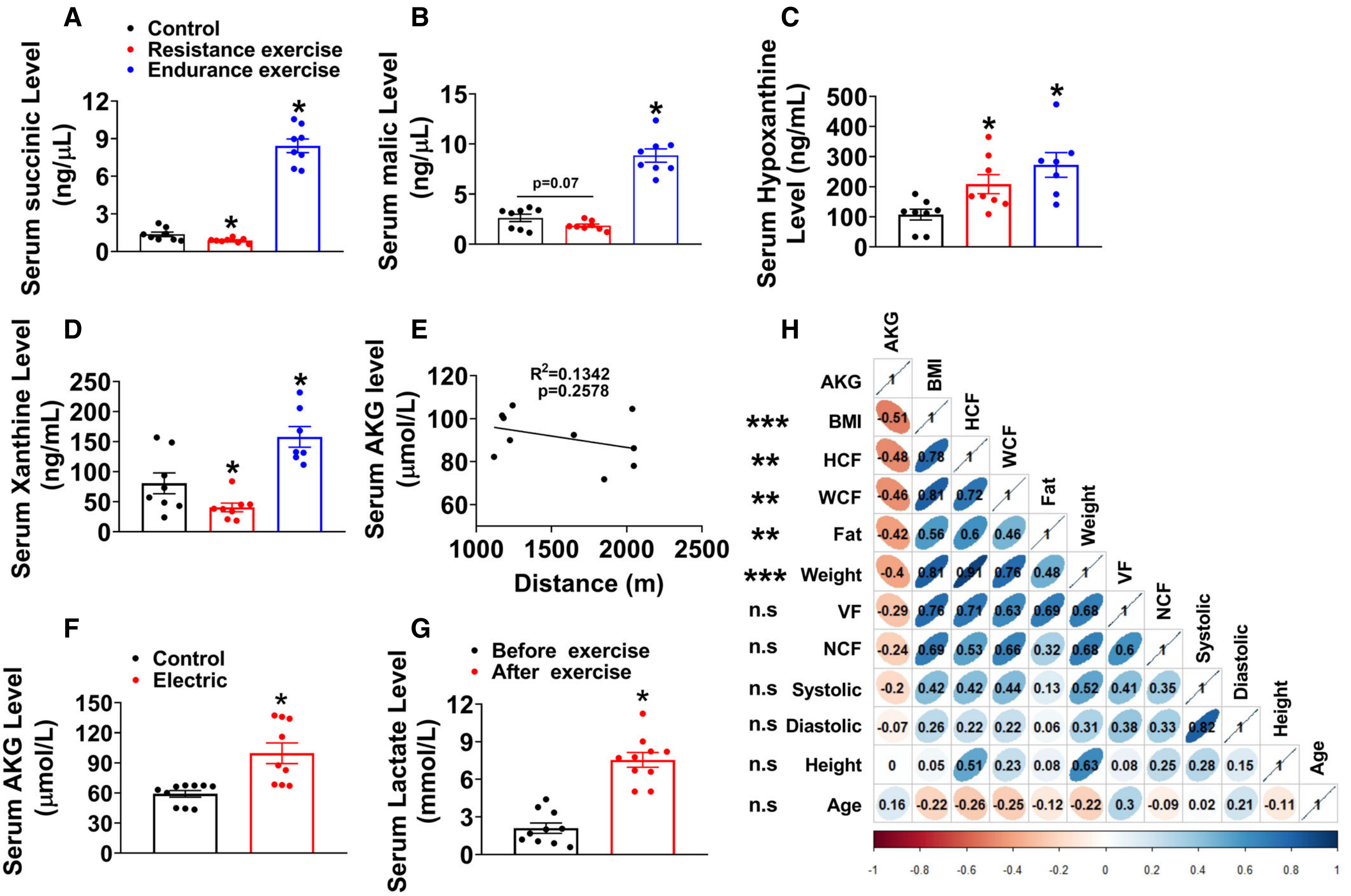

Figure EV1. Physiological relevance of AKG.

A–D The serum concentration of succinic acid (A), malic acid (B), hypoxanthine (C), and xanthine (D) in mice. Chow-fed male C57BL/6 mice (10 weeks old) were divided into three groups receiving non-exercise, endurance exercise (treadmill, 10 m/min, increased by 2 m/min every 2 min to exhaustion), or resistance exercise (ladder-climbing for 40 min) (n = 8 per group).

E Two-tailed Pearson's correlation coefficient analysis of plasma AKG level and the running distance of wheels. Chow-fed male C57BL/6 mice (10 weeks old) received 1-day free access to running wheel (n = 10 per group).

F Serum AKG level after electrical stimulation. Electrical stimulation was performed in unilateral gastrocnemius for 40 min (1 ms width/50 Hz, 10 times, each time for 4 min, resting for 2 min between stimulations) *in vivo* in 10-week-old male C57BL/6 mice fed with chow diet (n = 8-9 per group).

G Serum lactate concentration. Chow-fed male C57BL/6 mice (10 weeks old) received resistance exercise for 40 min. The serum lactate concentration was tested before and immediately after exercise (n = 10 per group).

H Two-tailed Pearson's correlation coefficient analysis of human plasma AKG level and body mass index (BMI), hip circumference (HCF), waist circumference (WCF), fat mass, body weight, visceral fat (VF), neck circumference (NCF), systolic pressure, diastolic pressure, height, and age. $^{**}P \leq 0.01$ and $^{***}P \leq 0.001$ indicate significant correlation between human plasma AKG level and BMI, HCF, WCF, fat mass, and body weight by one-way ANOVA followed by post hoc Tukey's tests. Red: negative correlation; blue: positive correlation.

Data information: Results are presented as mean ± SEM. In (A–D and G), $^{*}P \leq 0.05$ by one-way ANOVA followed by post hoc Tukey's tests.

© 2020 The Authors

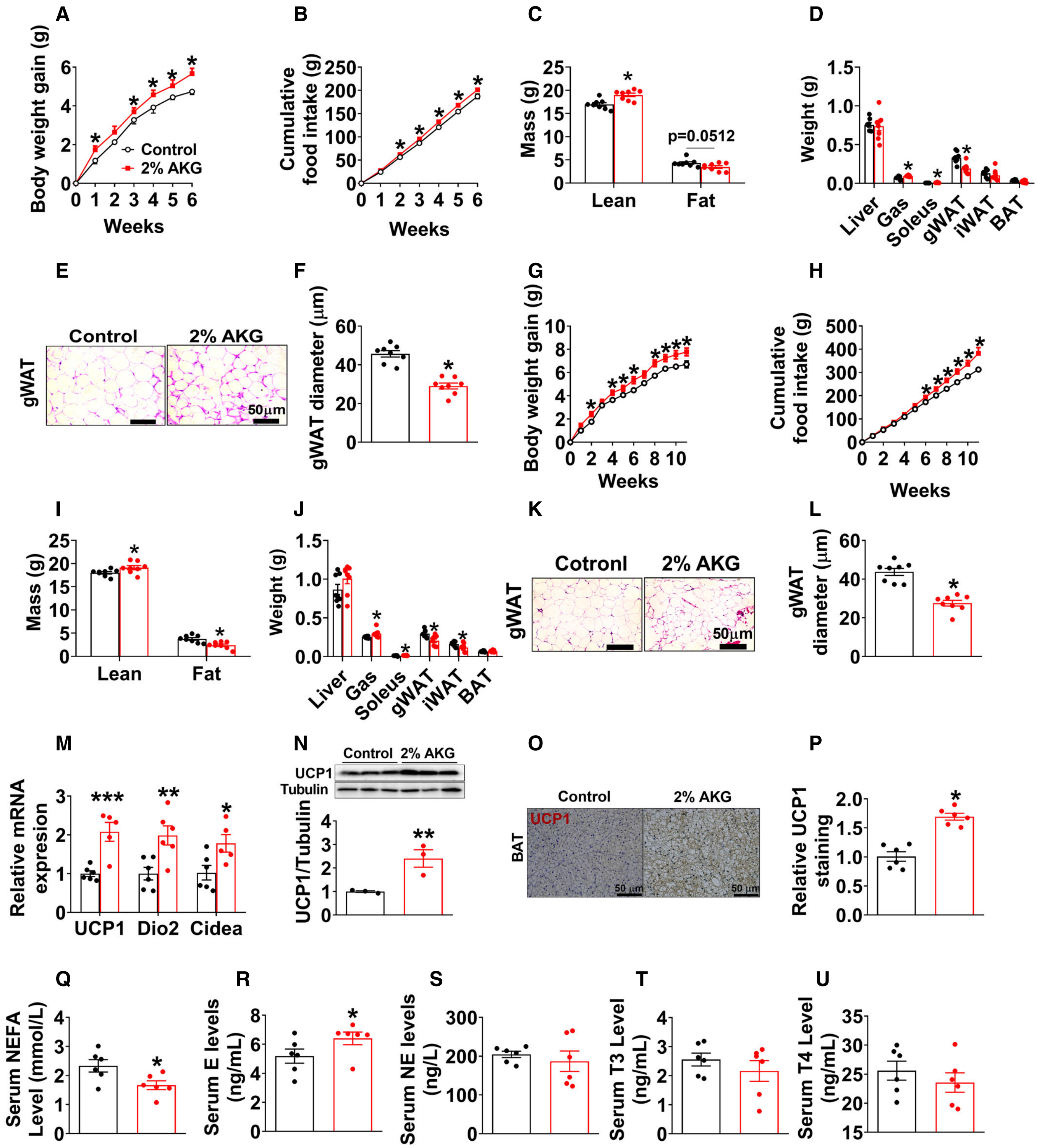

Figure EV2.

 © 2020 The Authors

Figure EV2. Metabolic effects of AKG in mice fed on chow.

A, B Body weight gain (A) and cumulative food intake (B) of male C57BL/6 mice. At 12 weeks of age, chow-fed male mice were divided into two groups receiving tap water or water supplemented with 2% AKG for 6 weeks (n = 8 per group).
C, D Body composition (C) and tissue weight (D) of male mice treated with AKG for 6 weeks (n = 7–8 per group).
E, F Representative images (E) and quantification (F) of gWAT HE staining from male mice treated with AKG for 6 weeks (n = 8 per group).
G, H Body weight gain (G) and cumulative food intake (H) of female C57BL/6 mice. At 12 weeks of age, chow-fed female mice were divided into two groups receiving tap water or water supplemented with 2% AKG for 11 weeks (n = 8 per group).
I, J Body composition (I) and tissue weight (J) of female mice treated with AKG for 11 weeks (n = 8 per group).
K, L Representative images (K) and quantification (L) of gWAT HE staining from female mice treated with AKG for 11 weeks (n = 8 per group).
M The mRNA expression of thermogenic genes in BAT of male C57BL/6 mice supplemented with AKG for 6 weeks (n = 6 per group).
N–P Immunoblots and quantification of UCP1 (N) and representative images of DAB staining (O) and quantification (P) of UCP1 in BAT of male mice supplemented with AKG for 6 weeks (n = 3–6 per group).
Q–U Serum levels of NEFA (Q), E (R), NE (S), T3 (T), and T4 (U) in male mice supplemented with AKG for 6 weeks (n = 6 per group).

Data information: Results are presented as mean ± SEM. In (A, B, G and H), $^{*}P \leq 0.05$ by two-way ANOVA followed by post hoc Bonferroni tests. In (C, D, F, I, J, L–N and P–U), $^{*}P \leq 0.05$, $^{**}P \leq 0.01$, and $^{***}P \leq 0.01$ by non-paired Student's t-test.

Source data are available online for this figure.

© 2020 The Authors

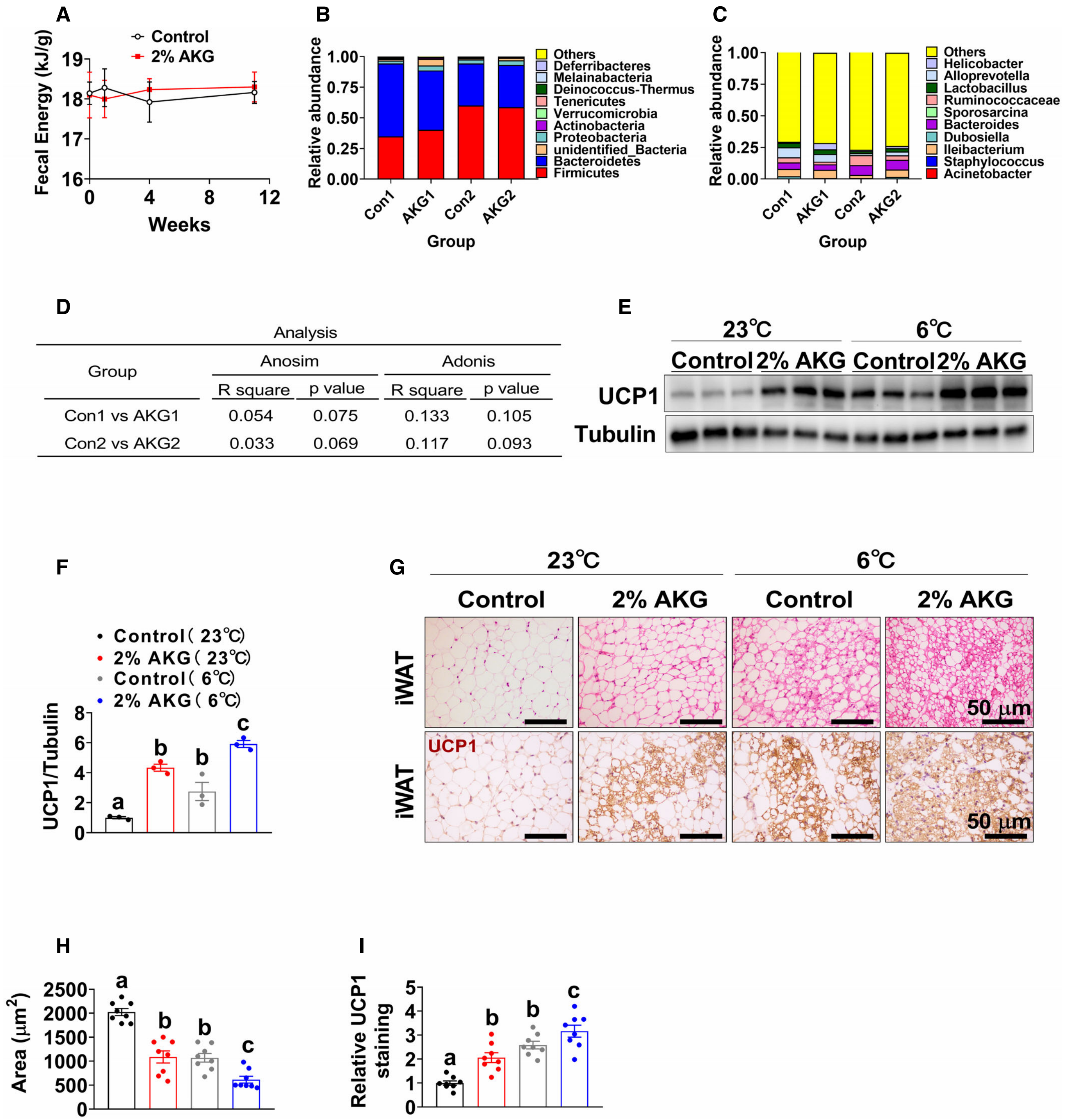

Figure EV3.

© 2020 The Authors

© 2020 The Authors

◀ **Figure EV3. Effects of AKG supplementation on fecal microbiota composition and iWAT browning in male mice.**

A Fecal energy of male C57BL/6 mice after 1, 4, and 11 weeks of AKG supplementation. At 12 weeks of age, male C57BL/6 mice were switched to HFD and received tap water or water supplemented with 2% AKG for 11 weeks (*n* = 9 per group).

B, C The fecal microbial composition in the phylum (B) and genus (C) in male C57BL/6 mice receiving 2% AKG water supplementation for 1 weeks (AKG1) or 4 weeks (AKG2) (*n* = 5 per group).

D Community structure test by ANOSIM and ADONIS of beta diversity in the genus between groups.

E, F Immunoblots (E) and quantification (F) of UCP1 protein in the iWAT of male C57BL/6 mice. At 10 weeks of age, male C57BL/6 mice were switched from chow to HFD and divided into four groups receiving tap water + room temperature (RT, 23°C), tap water + cold exposure (6°C), 2% AKG supplementation + 23°C, and 2% AKG supplementation + 6°C for one week (*n* = 8 per group).

G–I Representative images (G) and quantification (H, I) of HE staining or UCP1 staining in iWAT of male C57BL/6 mice (*n* = 8 per group).

Data information: Results are presented as mean ± SEM. In (A), data are analyzed by two-way ANOVA followed by post hoc Bonferroni tests. In (F, H and I), different letters between bars indicate $P \leq 0.05$ by one-way ANOVA followed by post hoc Tukey's tests.

© 2020 The Authors

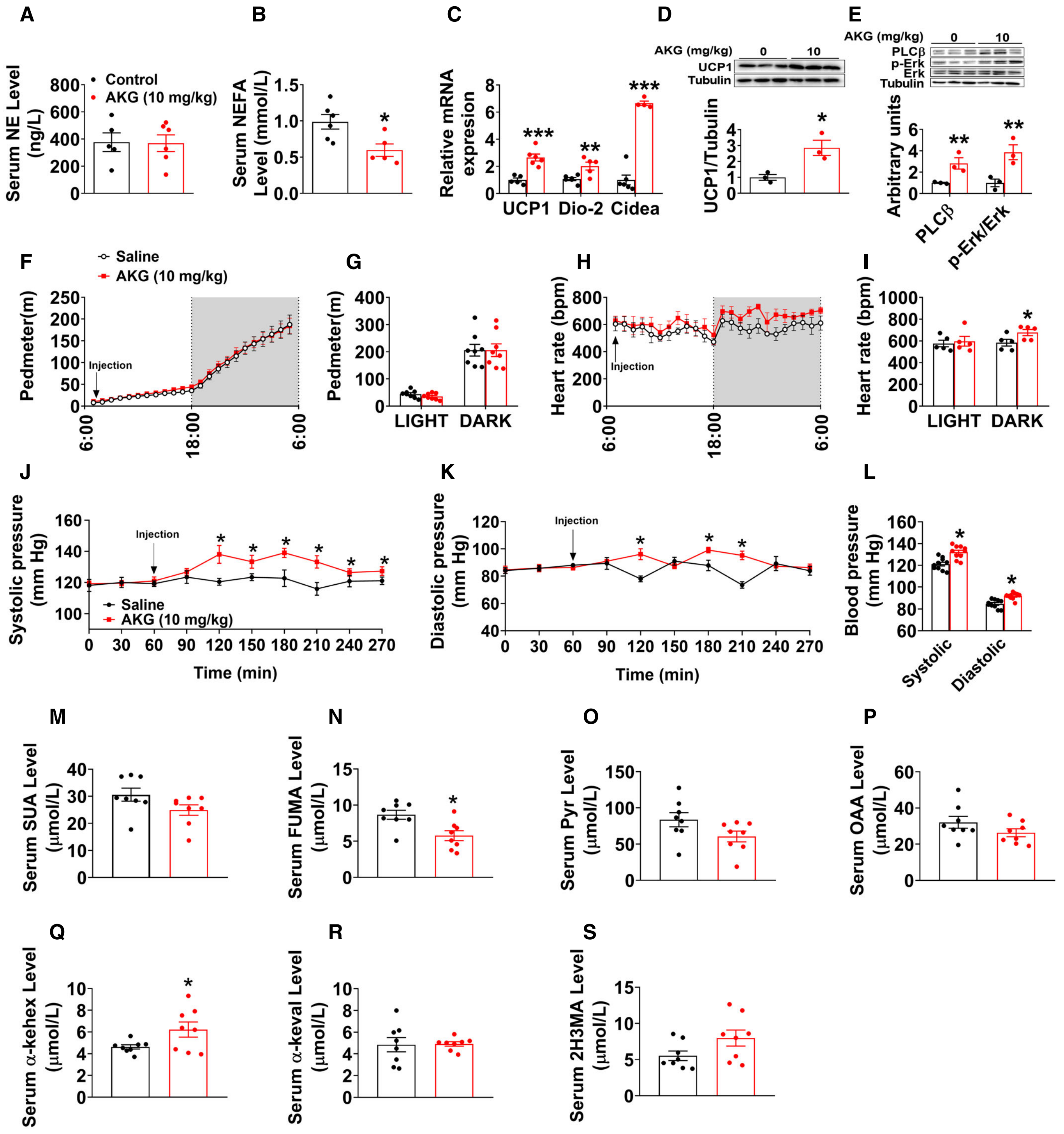

Figure EV4.

 © 2020 The Authors

36 of 30 The EMBO Journal e103304 | 2020 © 2020 The Authors

◀ **Figure EV4. Acute *in vivo* effects of AKG.**

A, B Serum levels of NE (A) and NEFA (B) in male C57BL/6 mice (10 weeks old) 3 h after i.p. injection of saline or AKG (10 mg/kg) (*n* = 5–6 per group).
C The mRNA expression of thermogenic genes in male C57BL/6 mice (10 weeks old) 3 h after i.p. injection of saline or AKG (10 mg/kg) (*n* = 5–6 per group).
D Immunoblots and quantification of UCP1 in BAT of male C57BL/6 mice (10 weeks old) 3 h after i.p. injection of saline or AKG (10 mg/kg) (*n* = 3 per group).
E Immunoblots and quantification of PLCβ and p-Erk in the adrenal glands of male C57BL/6 mice (10 weeks old) 3 h after i.p. injection of saline or AKG (10 mg/kg) (*n* = 3 per group).
F–I Physical activity (pedometer; F, G) and heart rate (H, I) of male mice i.p. injected with 10 mg/kg AKG or saline at 7:00 am (*n* = 8 per group).
J–L Blood pressure of male mice i.p. injected with 10 mg/kg AKG or saline (*n* = 8 per group).
M–S Serum levels of succinate (SUC) (M), fumaric acid (FUMA) (N), pyruvic acid (Pyr) (O), oxaloacetic acid (OAA) (P), α-ketoleucine (α-kehex) (Q), alpha-ketoisovaleric acid (α-keval) (R), and 2-hydroxy-3-methylbutyric acid (2H3MA) (S) in male C57BL/6 mice (10 weeks old) 3 h after i.p. injection of saline or AKG (10 mg/kg) (*n* = 8 per group).

Data information: Results are presented as mean ± SEM. In (A–E, G, I, L, N and Q), $*P \leq 0.05$, $**P \leq 0.01$, and $***P \leq 0.01$ by non-paired Student's *t*-test. In (F, H, J and K), $*P \leq 0.05$ by two-way ANOVA followed by post hoc Bonferroni tests.

© 2020 The Authors

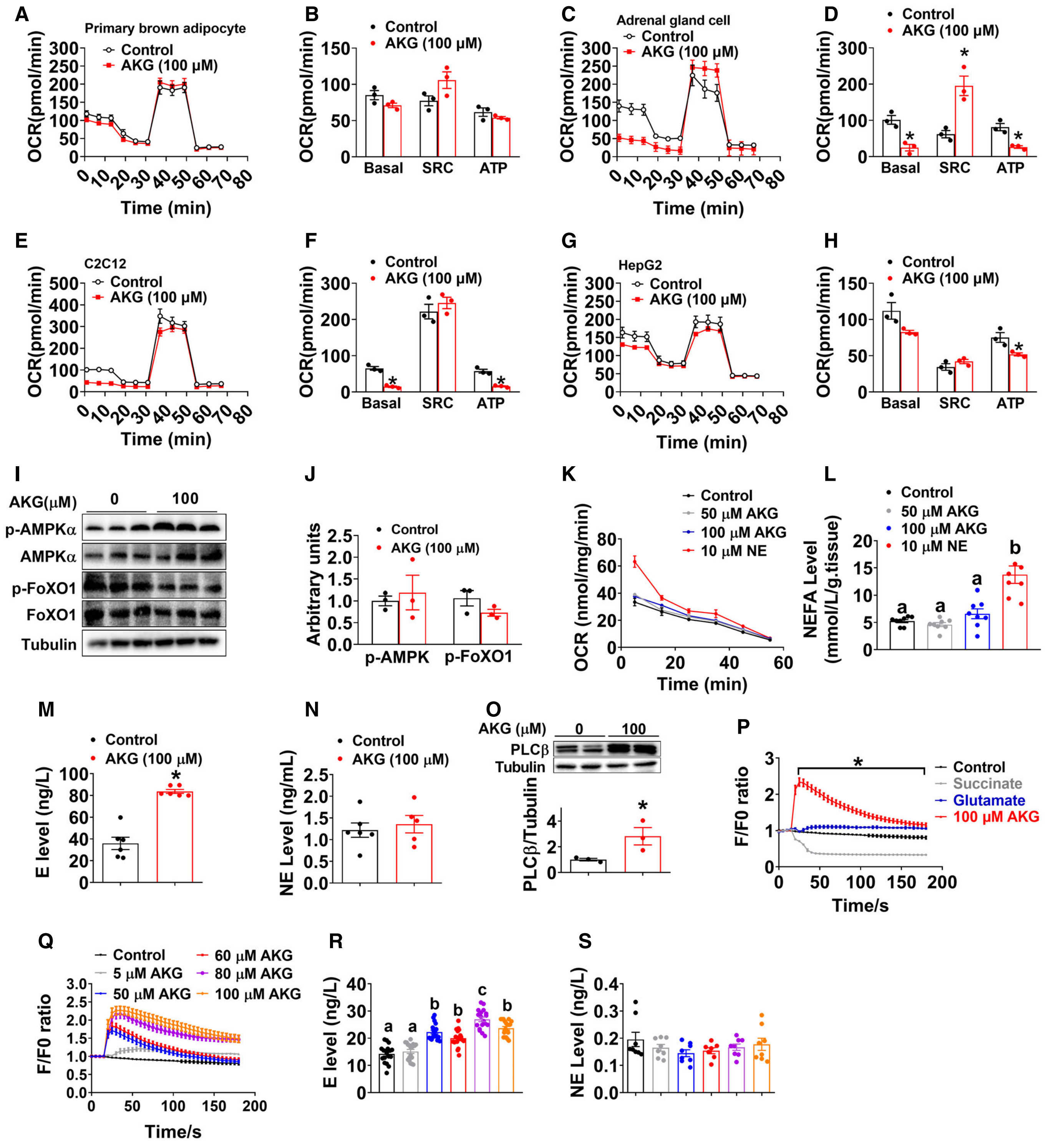

Figure EV5.

 © 2020 The Authors

 © 2020 The Authors

Figure EV5. Metabolic effects of AKG in *in vitro* and *ex vivo* models of BAT and adrenal gland.

A–H Oxygen consumption rate (OCR) of primary brown adipocyte (A, B), adrenal chromaffin cell line (C, D), C2C12 cell line (E, F), and HepG2 cell line (G, H) treated with vehicle or 100 μM AKG for 3 h (*n* = 3 per group). OCR was monitored using the Agilent Seahorse XFp analyzer with the sequential injection of oligomycin, FCCP, and rotenone/antimycin.

I, J Immunoblots (I) and quantification (J) of p-AMPKα and p-FoxO1 in primary brown adipocytes treated with vehicle or 100 μM AKG (*n* = 3 per group).

K Oxygen consumption rate (OCR) of *ex vivo* BAT cultured with vehicle, 50 μM AKG, 100 μM AKG, or 10 μM NE for 5, 15, 25, 25, 45, and 55 min (*n* = 3 per group).

L Medium NEFA level from *ex vivo* BAT treated with vehicle, 50 μM AKG, 100 μM AKG, or 10 μM NE for 30 min (*n* = 6 per group).

M, N Medium E (M) and NE (N) levels from *ex vivo* adrenal gland treated with vehicle or 100 μM AKG for 30 min *in vitro* (*n* = 5–6 per group).

O Immunoblots and quantification of PLCβ in *ex vivo* adrenal gland treated with vehicle or 100 μM AKG for 30 min *in vitro* (*n* = 3 per group).

P Intracellular calcium ion [Ca^{2+}] changes in *in vitro* adrenal chromaffin cells treated with vehicle, 100 μM AKG, 100 μM succinate, or 100 μM glutamine (*n* = 30 per group).

Q Intracellular calcium ion [Ca^{2+}] changes in *in vitro* adrenal chromaffin cells treated with vehicle or 5, 50, 60, 80, or 100 μM AKG (*n* = 30 per group).

R, S Medium E (R) or NE (S) level from *in vitro* adrenal chromaffin cells treated with vehicle or 5, 50, 60, 80, or 100 μM AKG for 30 min (*n* = 8–18 per group).

Data information: Results are presented as mean ± SEM. In (B, D, F, H, J, and M–O), *$P \leq 0.05$ by non-paired Student's *t*-test. In (K and P–Q), *$P \leq 0.05$ by two-way ANOVA followed by post hoc Bonferroni tests. In (L and R–S), different letters indicate significant differences between groups by one-way ANOVA followed by post hoc Tukey's tests.

Source data are available online for this figure.
