# Supplementary figures and images for: Exercise‐induced α‐ketoglutaric acid stimulates muscle hypertrophy and fat loss through OXGR1‐dependent adrenal activation
Source: EMBO J. 2020 Feb 27;39(7):e103304. doi: 10.15252/embj.2019103304 (PMC7110140; doi:10.15252/embj.2019103304)

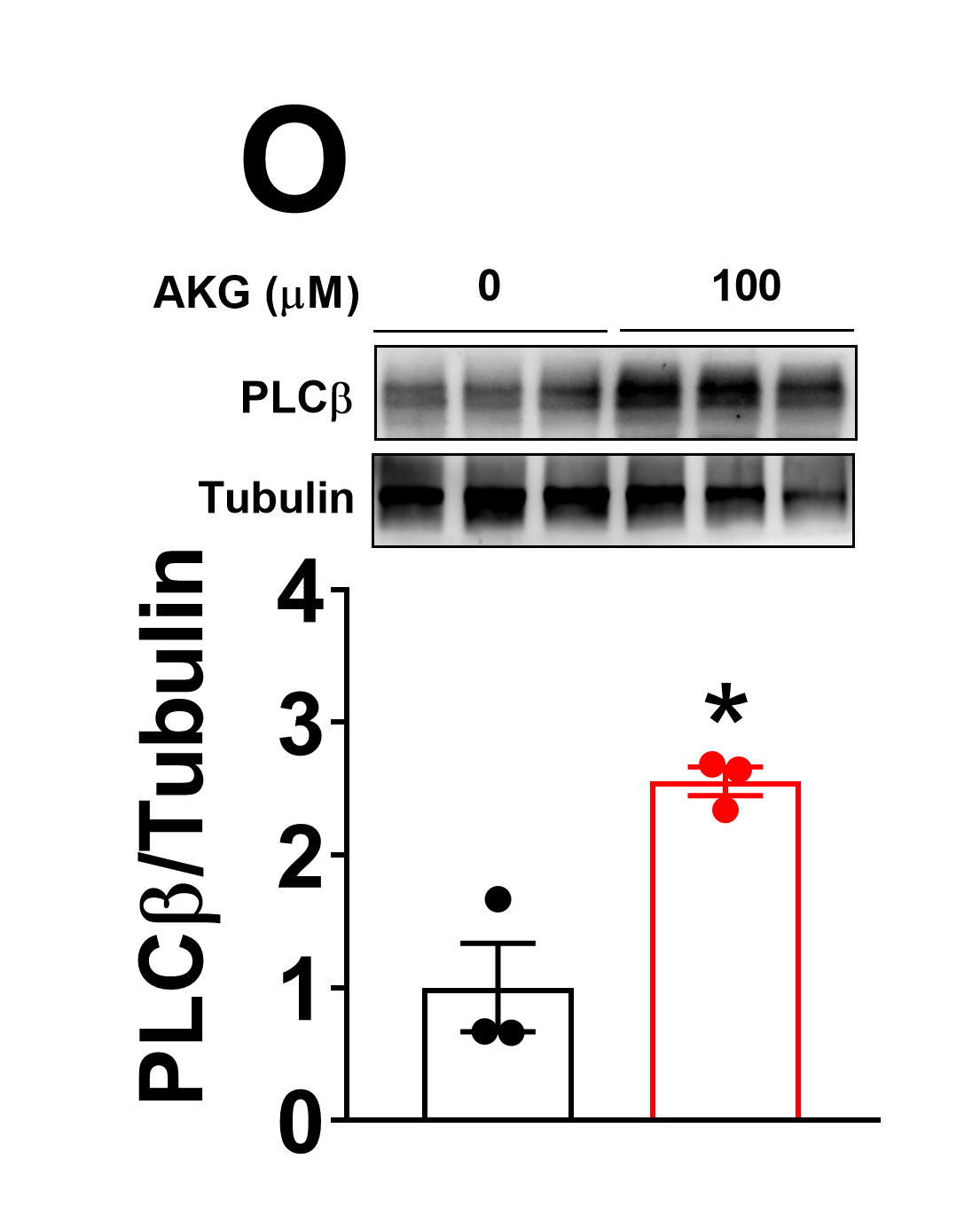

Supplement: Supplementary file 3 — Source Data for Expanded View [file EMBJ-39-e103304-s005.zip › embj2019103304-sup-0005-SDataFigEV/Figure_EV5O-New.tif]

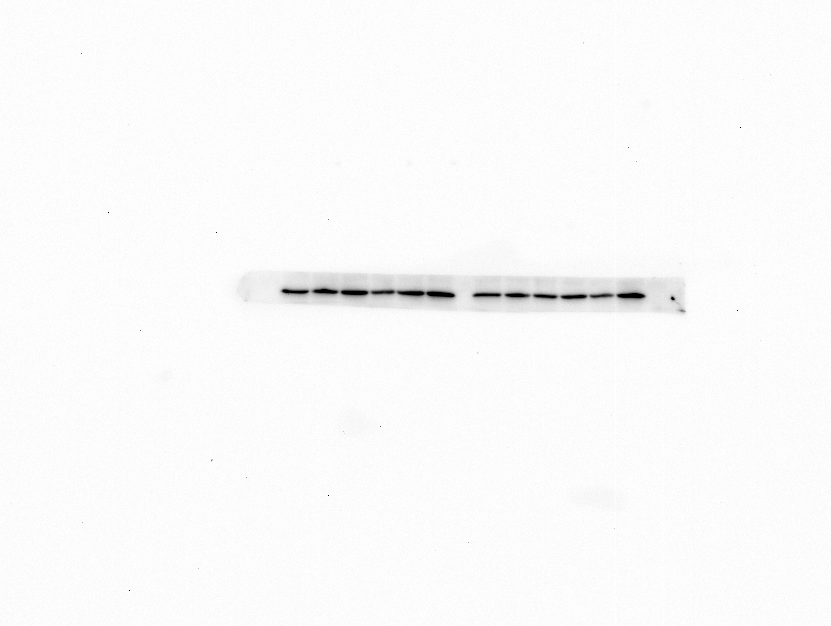

Supplement: Supplementary file 3 — Source Data for Expanded View [file EMBJ-39-e103304-s005.zip › embj2019103304-sup-0005-SDataFigEV/Fig_EV2N-Tubulin_(n=3)(Lane7-12)(representative_picture).png]

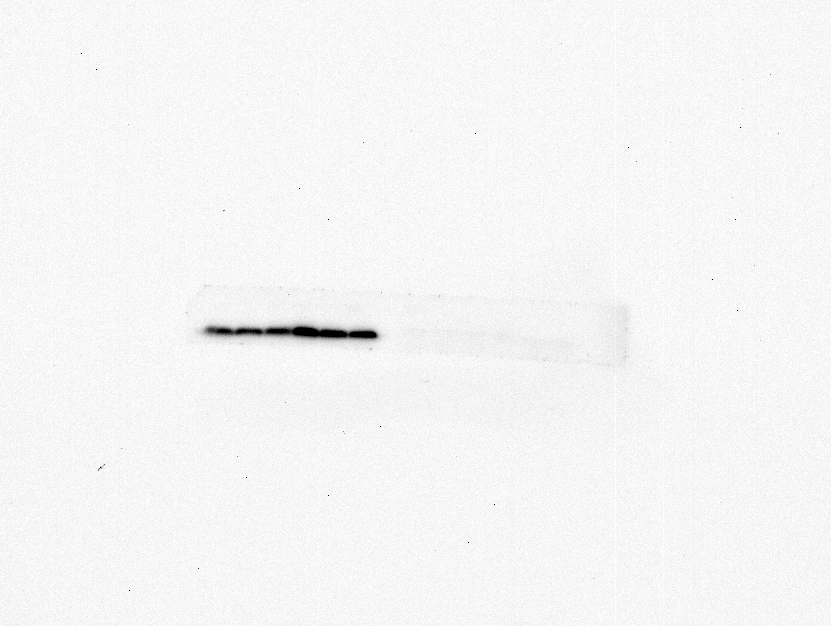

Supplement: Supplementary file 3 — Source Data for Expanded View [file EMBJ-39-e103304-s005.zip › embj2019103304-sup-0005-SDataFigEV/Fig_EV2N-UCP1_(n=3)(Lane1-6)(representative_picture).png]

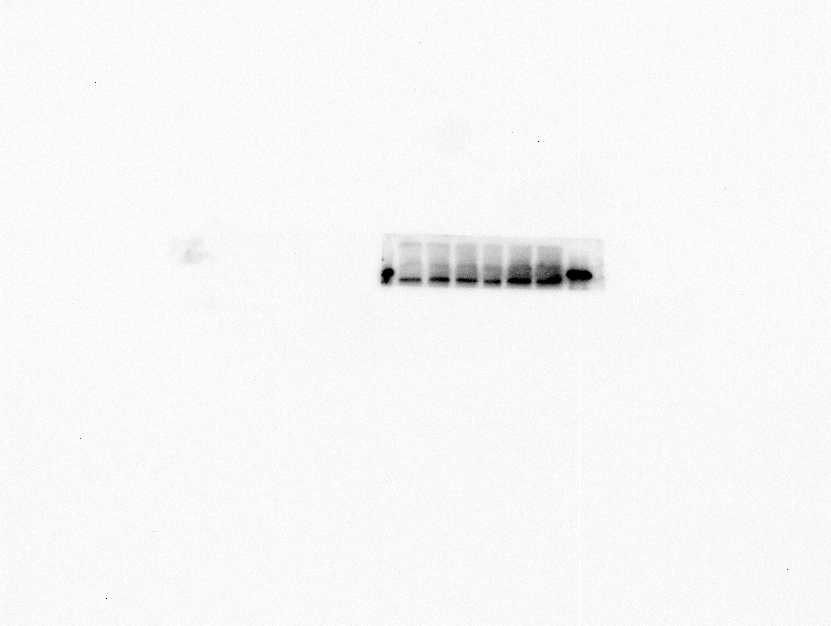

Supplement: Supplementary file 3 — Source Data for Expanded View [file EMBJ-39-e103304-s005.zip › embj2019103304-sup-0005-SDataFigEV/Fig_EV5I-AMPKα_(n=3)(Lane1-6)(representative_picture).png]

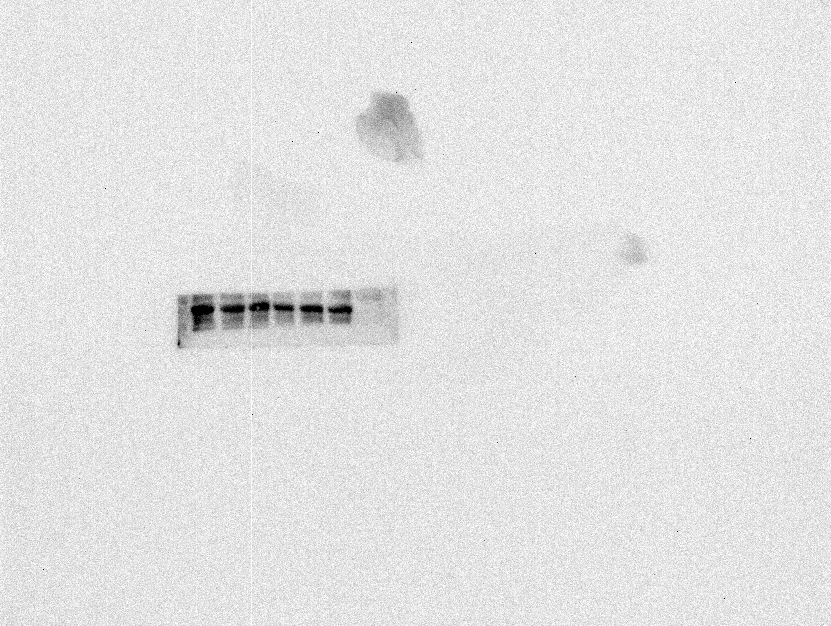

Supplement: Supplementary file 3 — Source Data for Expanded View [file EMBJ-39-e103304-s005.zip › embj2019103304-sup-0005-SDataFigEV/Fig_EV5I-FoXO1(n=3)(Lane1-6)(representative_picture).png]

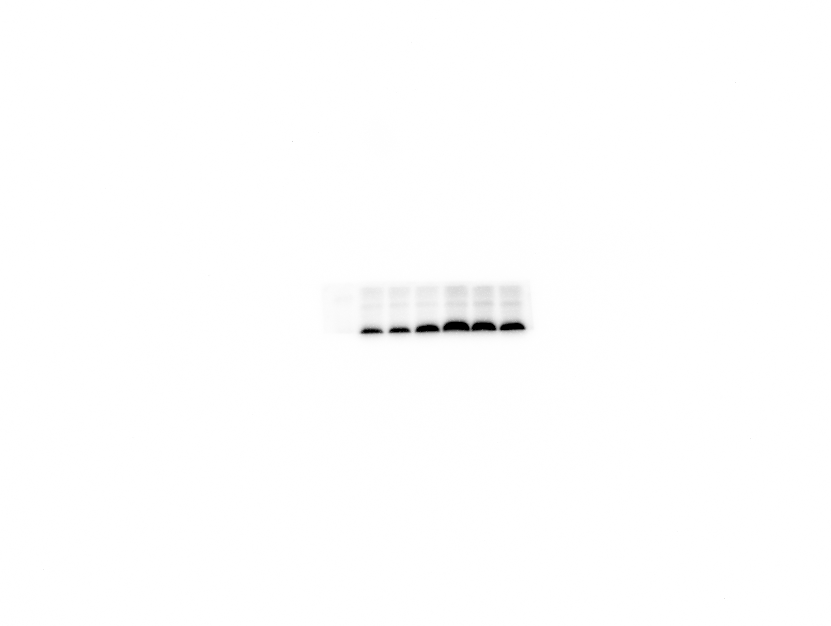

Supplement: Supplementary file 3 — Source Data for Expanded View [file EMBJ-39-e103304-s005.zip › embj2019103304-sup-0005-SDataFigEV/Fig_EV5I-p-AMPKα_(n=3)(Lane1-6)(representative_picture).png]

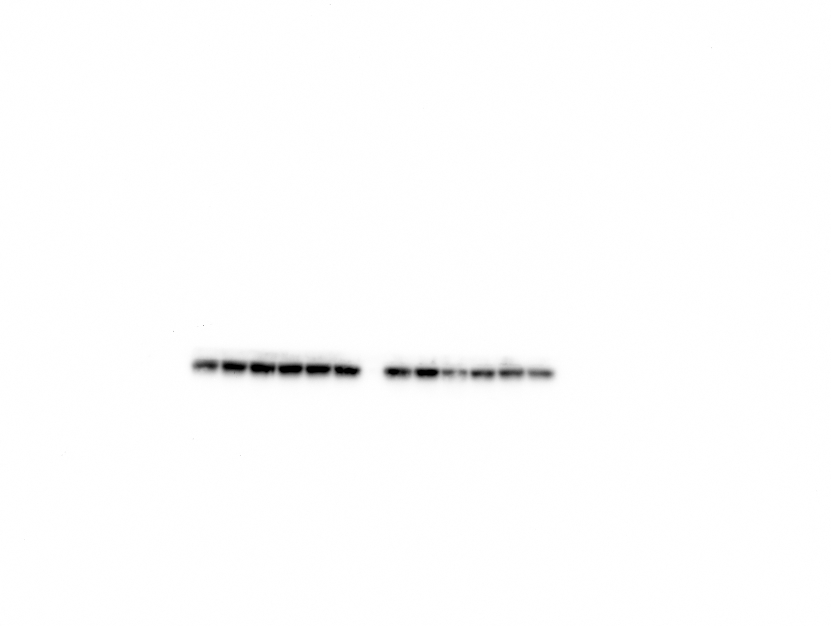

Supplement: Supplementary file 3 — Source Data for Expanded View [file EMBJ-39-e103304-s005.zip › embj2019103304-sup-0005-SDataFigEV/Fig_EV5I-Tubulin_(n=3)(Lane1-6)(representative_picture).png]

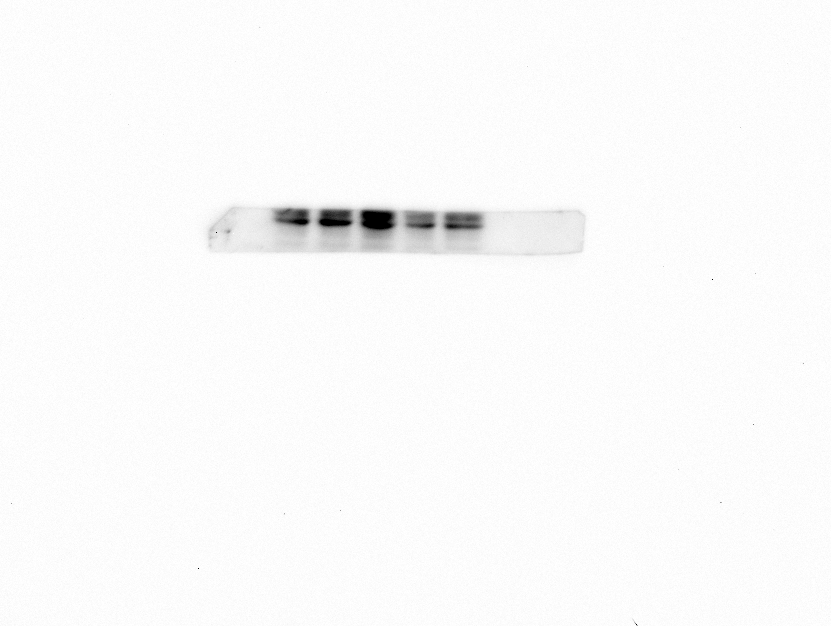

Supplement: Supplementary file 3 — Source Data for Expanded View [file EMBJ-39-e103304-s005.zip › embj2019103304-sup-0005-SDataFigEV/Fig_EV5O-PLC╬▓_(n=1)(Lane1-2)(2).png]

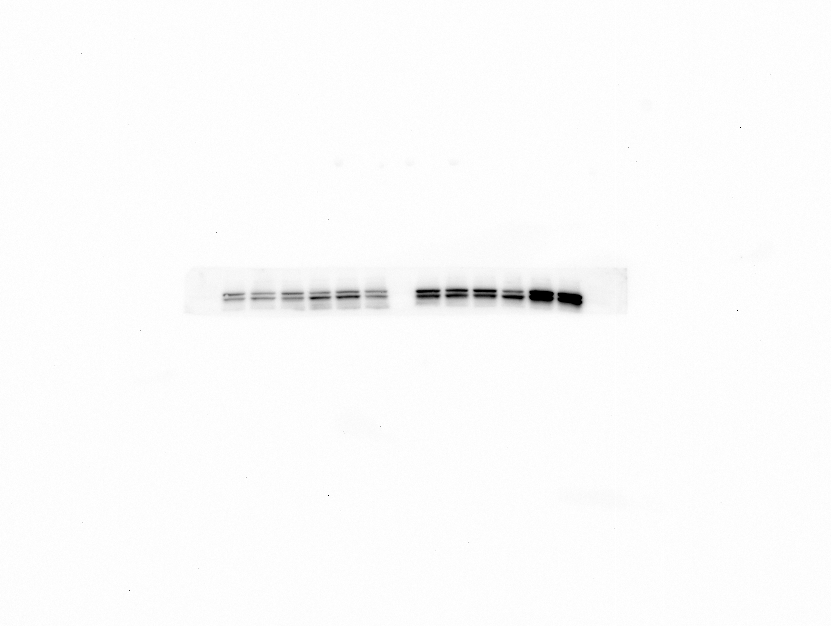

Supplement: Supplementary file 3 — Source Data for Expanded View [file EMBJ-39-e103304-s005.zip › embj2019103304-sup-0005-SDataFigEV/Fig_EV5O-PLC╬▓_(n=2)(Lane9-12)(representative_picture).png]

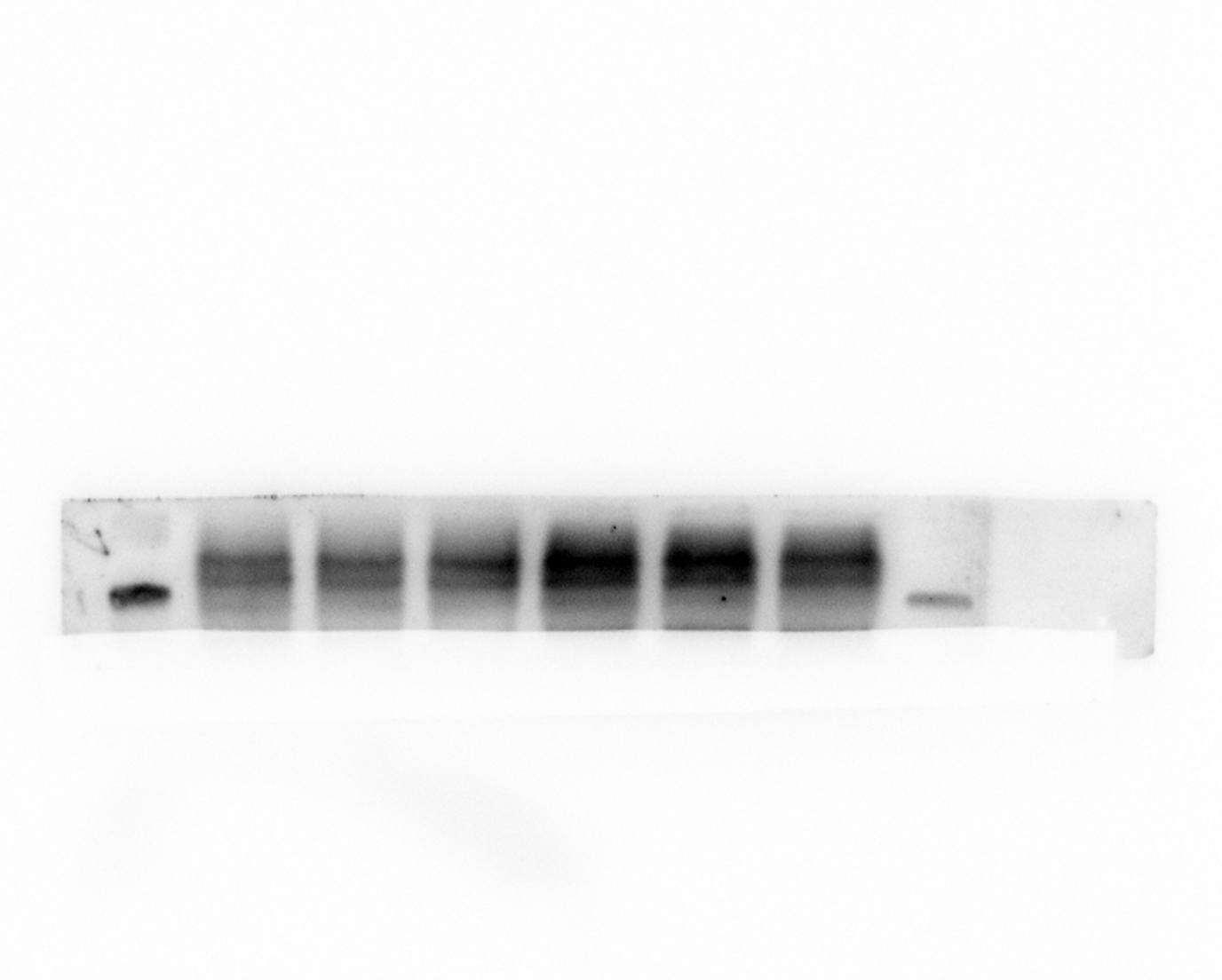

Supplement: Supplementary file 3 — Source Data for Expanded View [file EMBJ-39-e103304-s005.zip › embj2019103304-sup-0005-SDataFigEV/Fig_EV5O-PLC╬▓_(n=3)(Lane1-6)(representative_picture)-New.Tif]

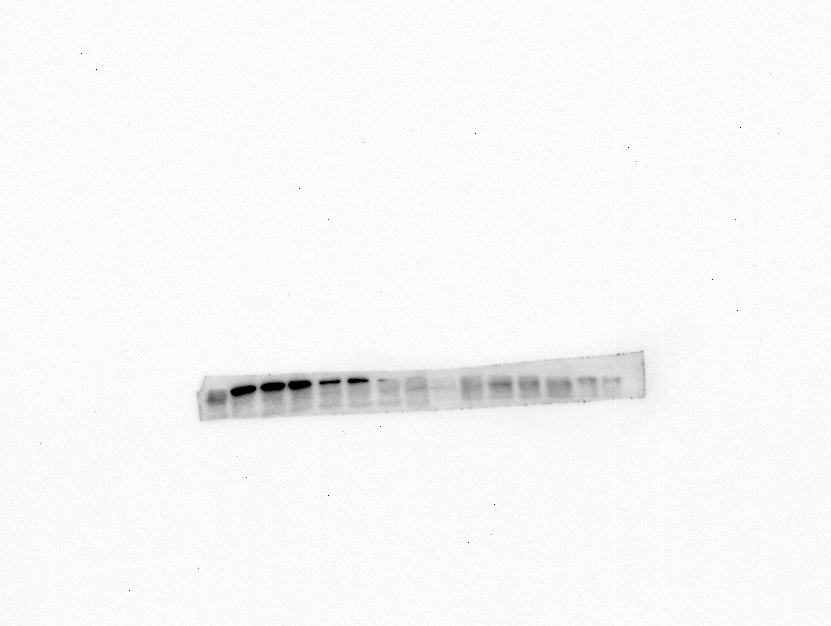

Supplement: Supplementary file 3 — Source Data for Expanded View [file EMBJ-39-e103304-s005.zip › embj2019103304-sup-0005-SDataFigEV/Fig_EV5O-Tubulin_(n=1)(Lane1-2)(2).png]

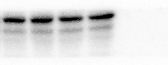

Supplement: Supplementary file 3 — Source Data for Expanded View [file EMBJ-39-e103304-s005.zip › embj2019103304-sup-0005-SDataFigEV/Fig_EV5O-Tubulin_(n=2)(Lane1-4)(representative_picture).tif]

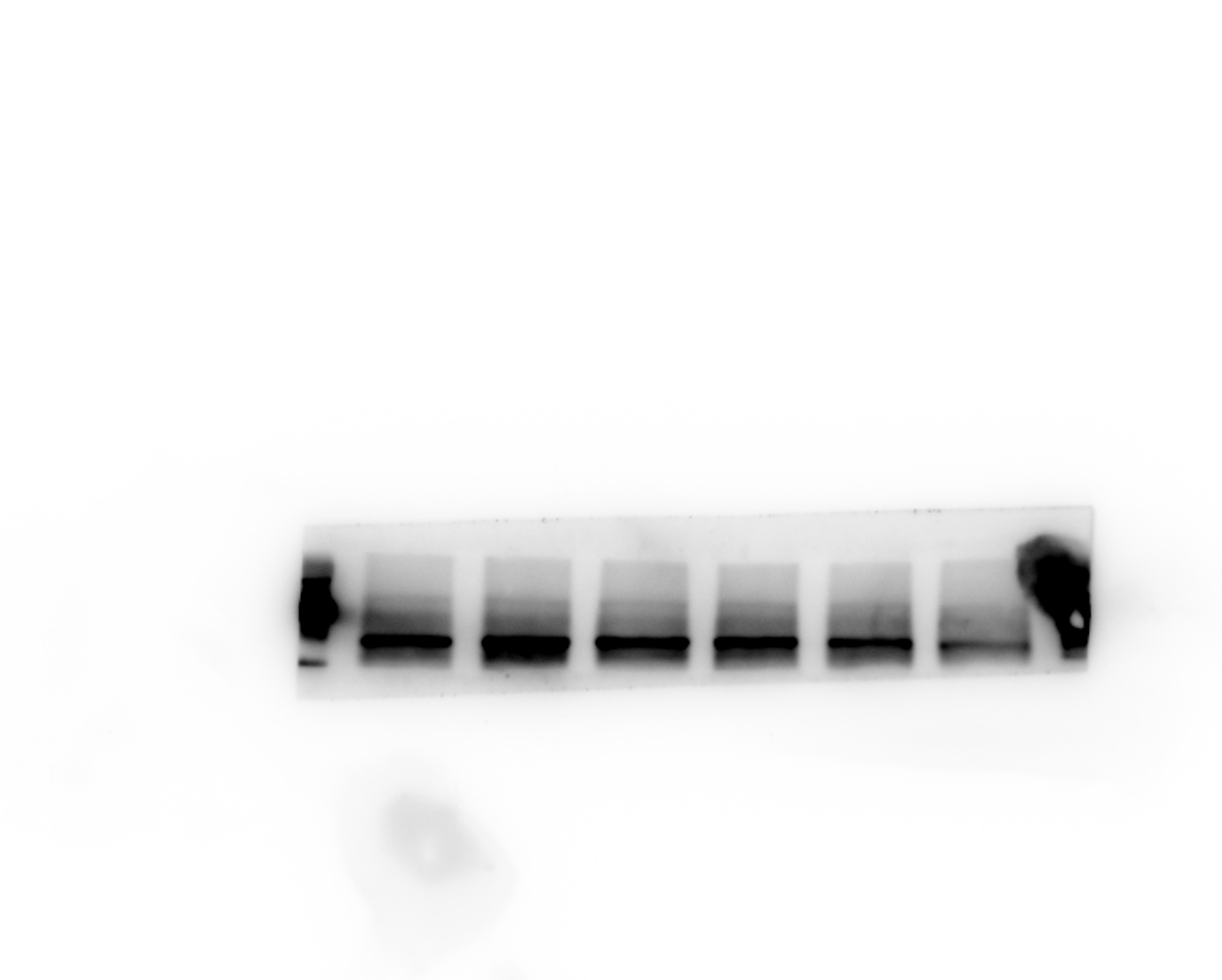

Supplement: Supplementary file 3 — Source Data for Expanded View [file EMBJ-39-e103304-s005.zip › embj2019103304-sup-0005-SDataFigEV/Fig_EV5O-Tubulin_(n=3)(Lane1-6)(representative_picture)-New.Tif]

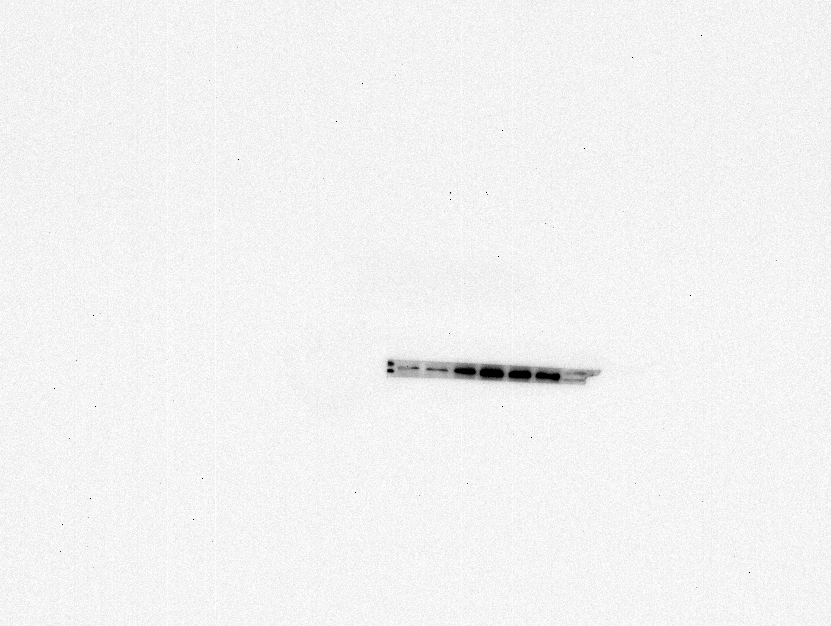

Supplement: Supplementary file 5 — Source Data for Figure 4 [file EMBJ-39-e103304-s003.zip › Fig_4D-ATGL_(n=3)(Lane1-6)(representative_picture).png]

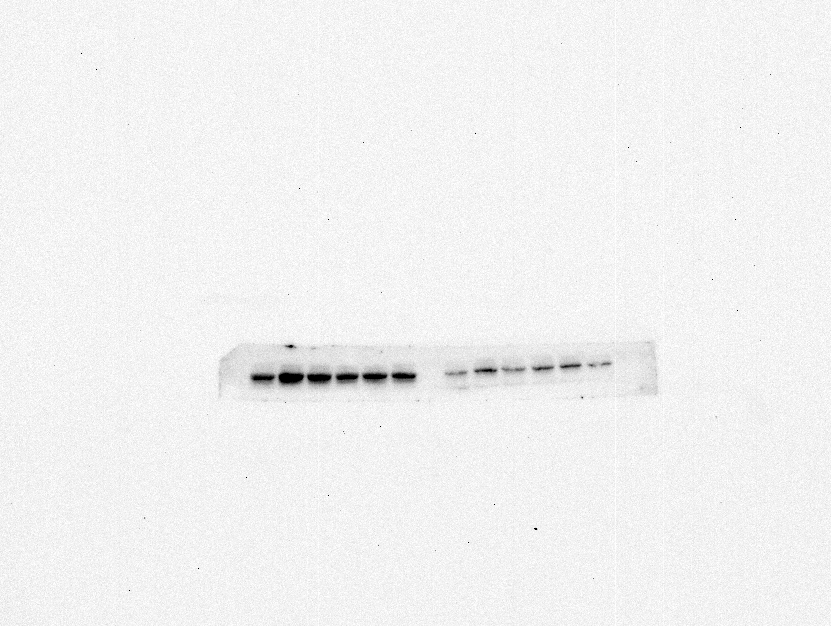

Supplement: Supplementary file 5 — Source Data for Figure 4 [file EMBJ-39-e103304-s003.zip › Fig_4D-HSL_(n=3)(Lane1-6)(representative_picture).png]

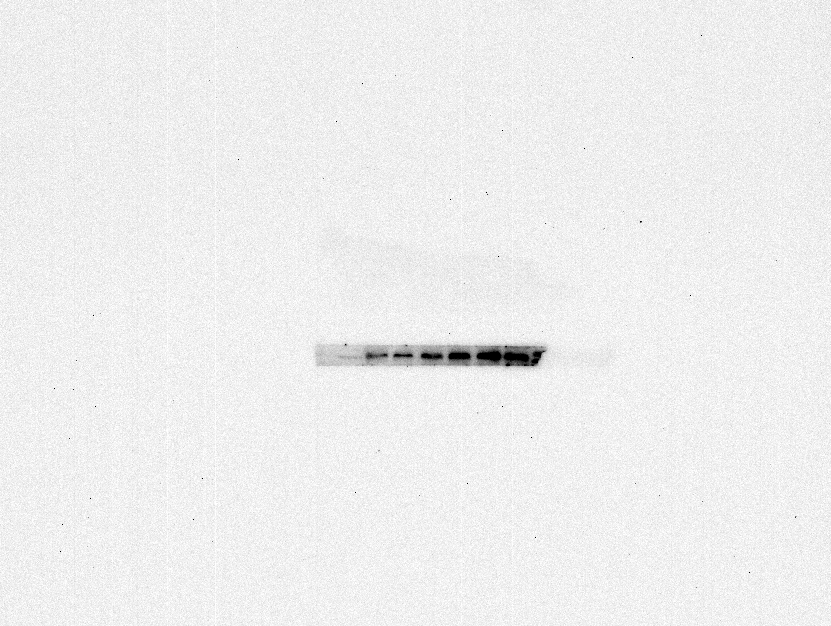

Supplement: Supplementary file 5 — Source Data for Figure 4 [file EMBJ-39-e103304-s003.zip › Fig_4D-p-HSL_(n=3)(Lane1-6)(representative_picture).png]

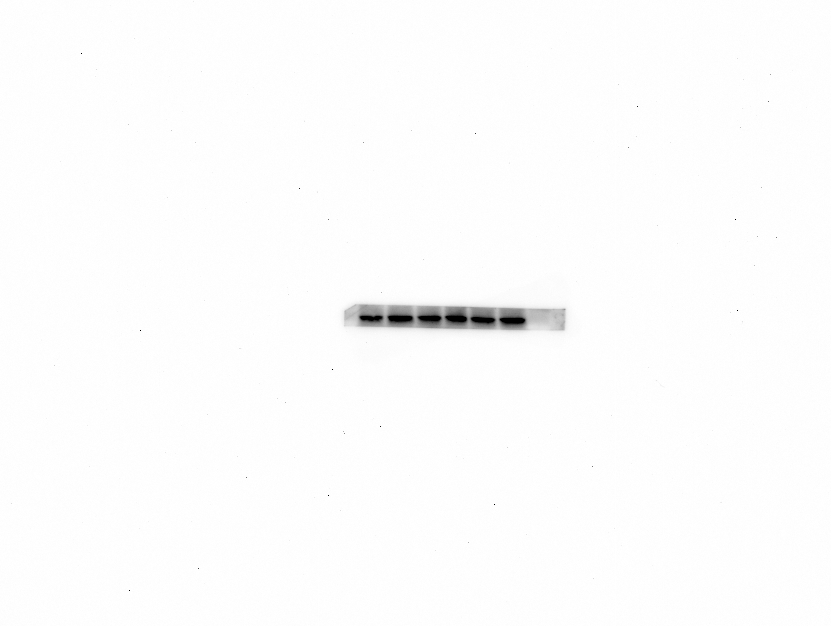

Supplement: Supplementary file 5 — Source Data for Figure 4 [file EMBJ-39-e103304-s003.zip › Fig_4D-Tubulin_(n=3)(Lane1-6)(representative_picture).png]

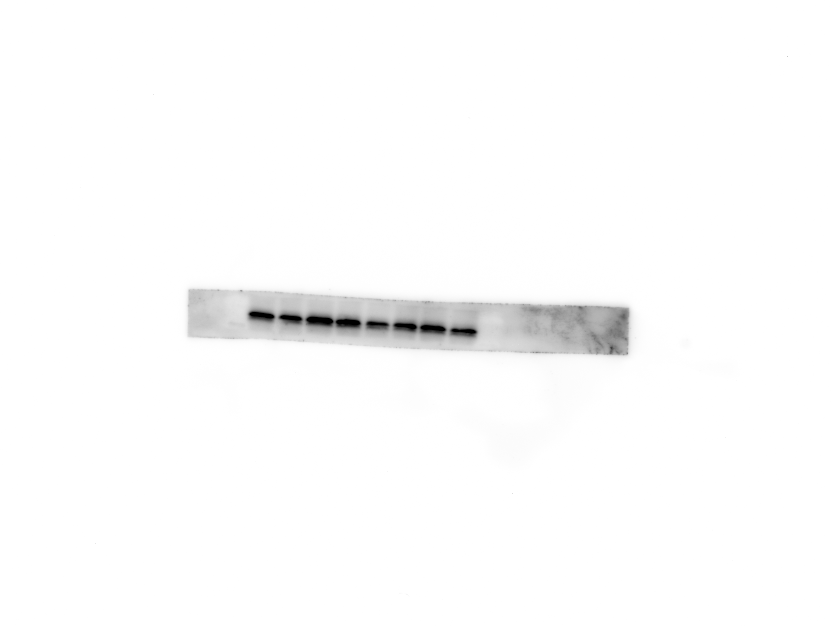

Supplement: Supplementary file 5 — Source Data for Figure 4 [file EMBJ-39-e103304-s003.zip › Fig_4R-ATGL(n=2)(Lane1-8)(2).png]

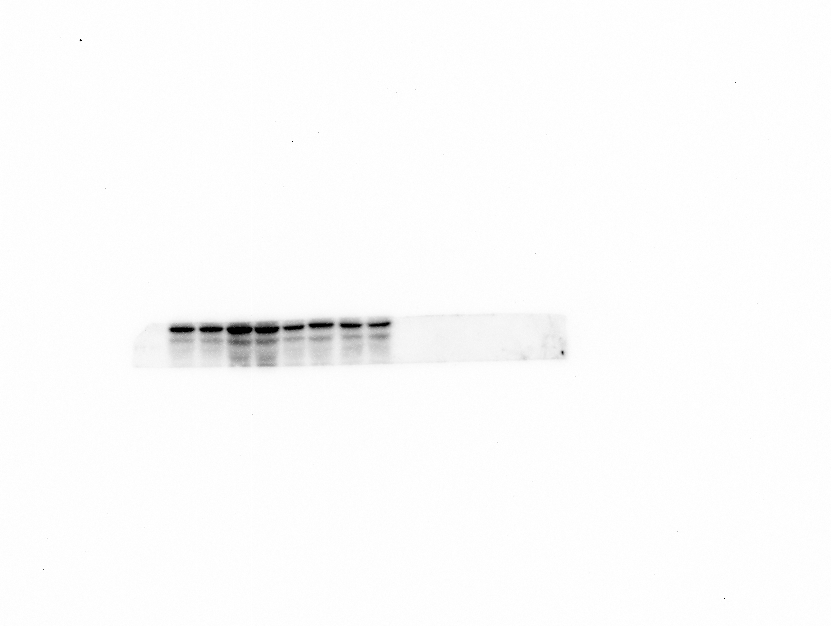

Supplement: Supplementary file 5 — Source Data for Figure 4 [file EMBJ-39-e103304-s003.zip › Fig_4R-ATGL_(n=2)(Lane1-8)(representative_picture).png]

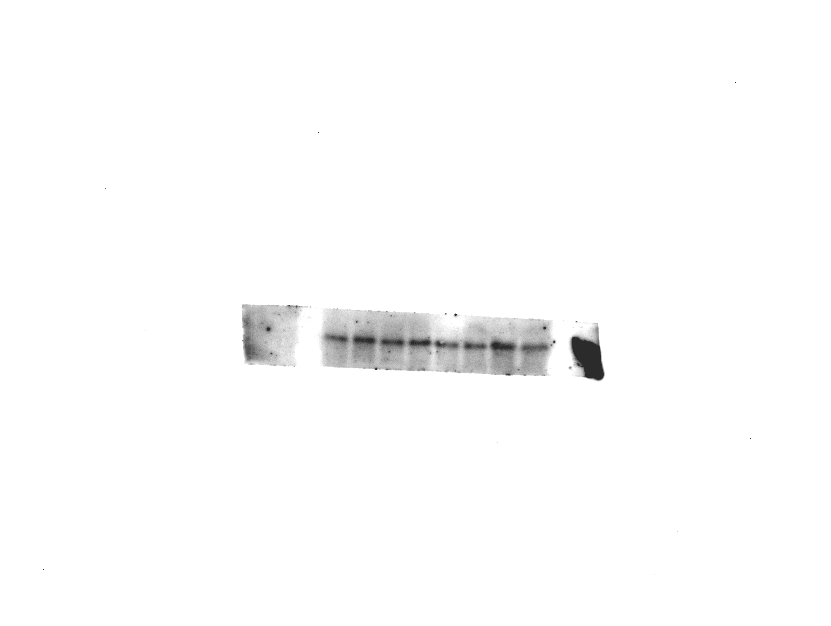

Supplement: Supplementary file 5 — Source Data for Figure 4 [file EMBJ-39-e103304-s003.zip › Fig_4R-HSL(n=2)(Lane1-8)(2).png]

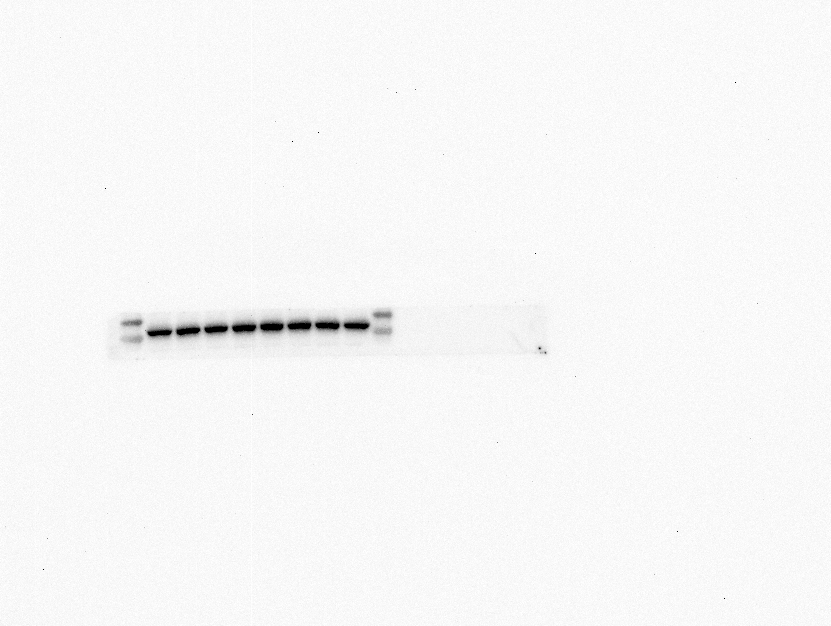

Supplement: Supplementary file 5 — Source Data for Figure 4 [file EMBJ-39-e103304-s003.zip › Fig_4R-HSL_(n=2)(Lane1-8)(representative_picture).png]

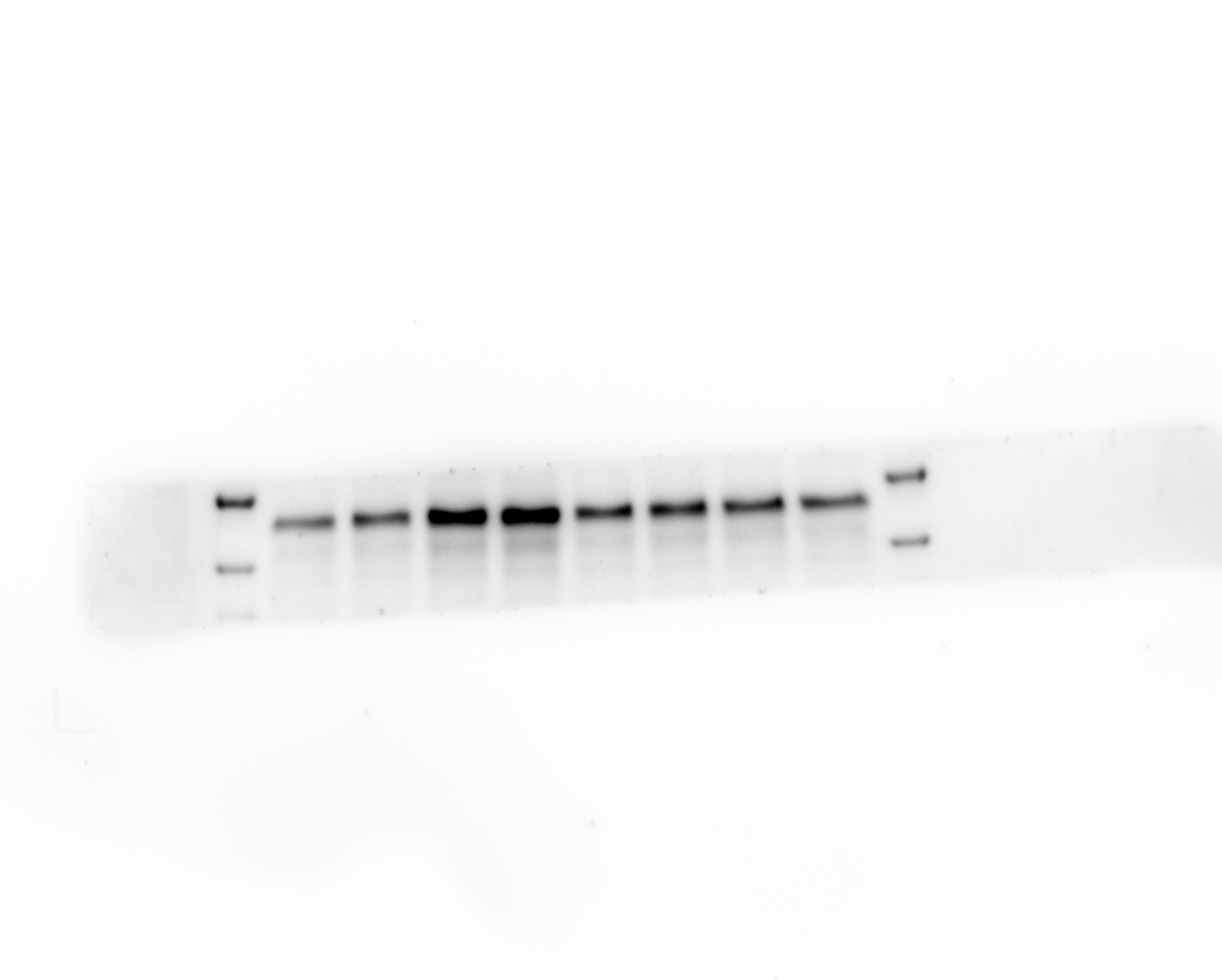

Supplement: Supplementary file 5 — Source Data for Figure 4 [file EMBJ-39-e103304-s003.zip › Fig_4R-p-HSL(n=2)(Lane1-8)(2).Tif]

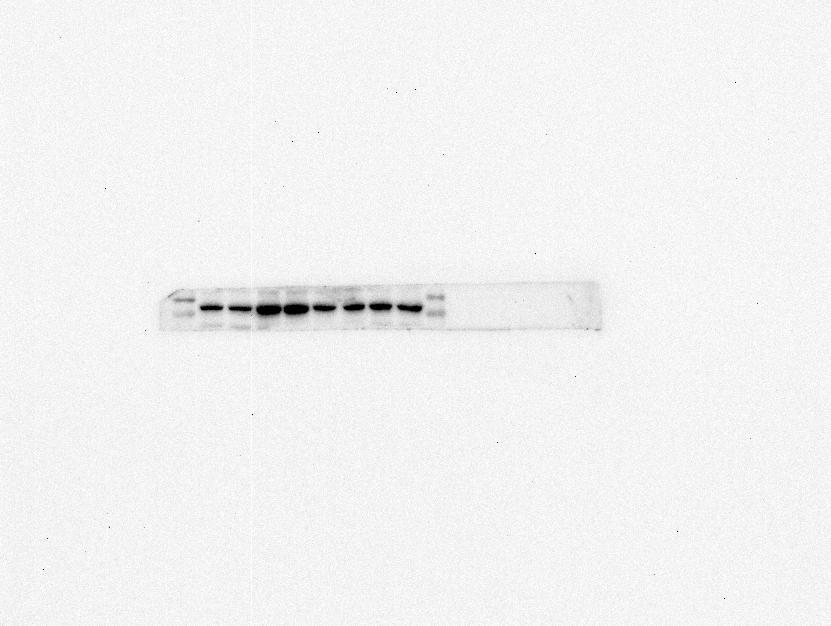

Supplement: Supplementary file 5 — Source Data for Figure 4 [file EMBJ-39-e103304-s003.zip › Fig_4R-p-HSL_(n=2)(Lane1-8)(representative_picture).png]

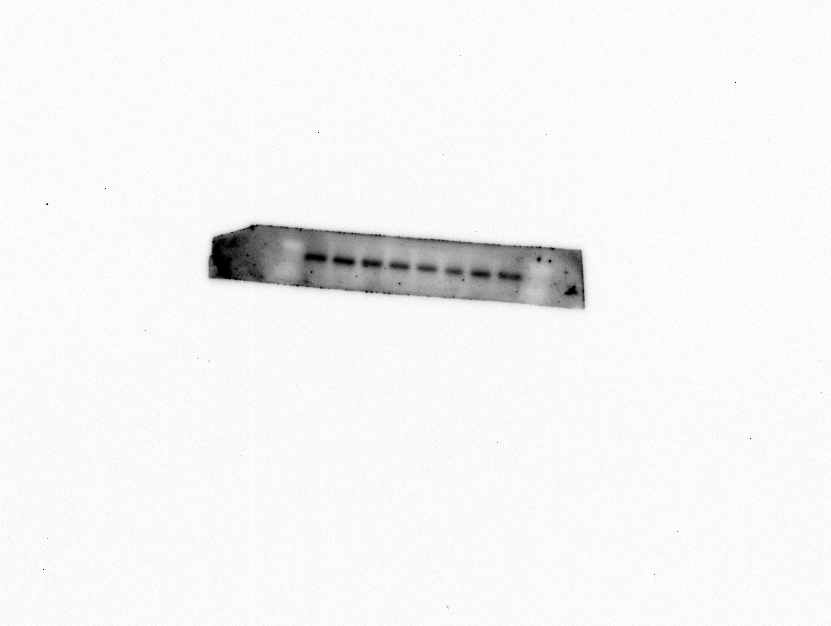

Supplement: Supplementary file 5 — Source Data for Figure 4 [file EMBJ-39-e103304-s003.zip › Fig_4R-Tubulin(n=2)(Lane1-8)(2).png]

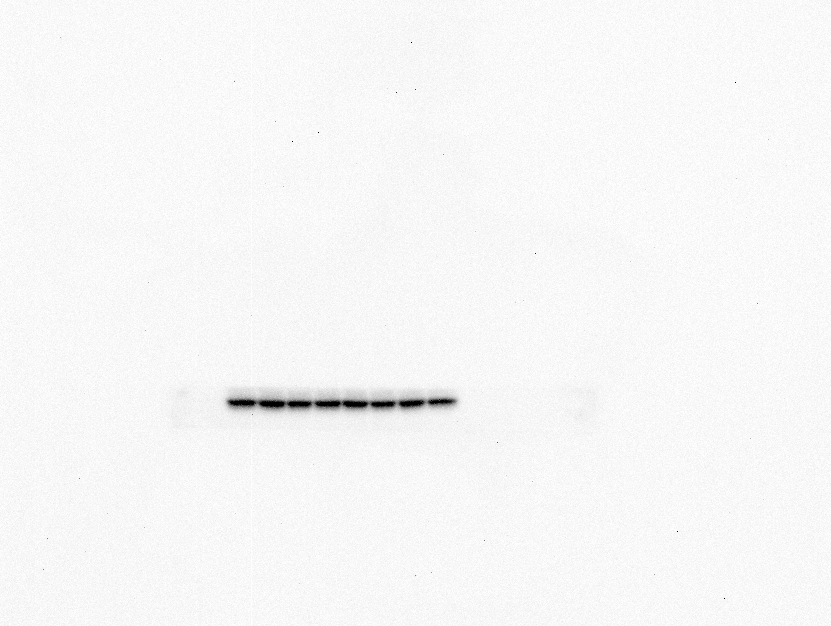

Supplement: Supplementary file 5 — Source Data for Figure 4 [file EMBJ-39-e103304-s003.zip › Fig_4R-Tubulin_(n=2)(Lane1-8)(representative_picture).png]

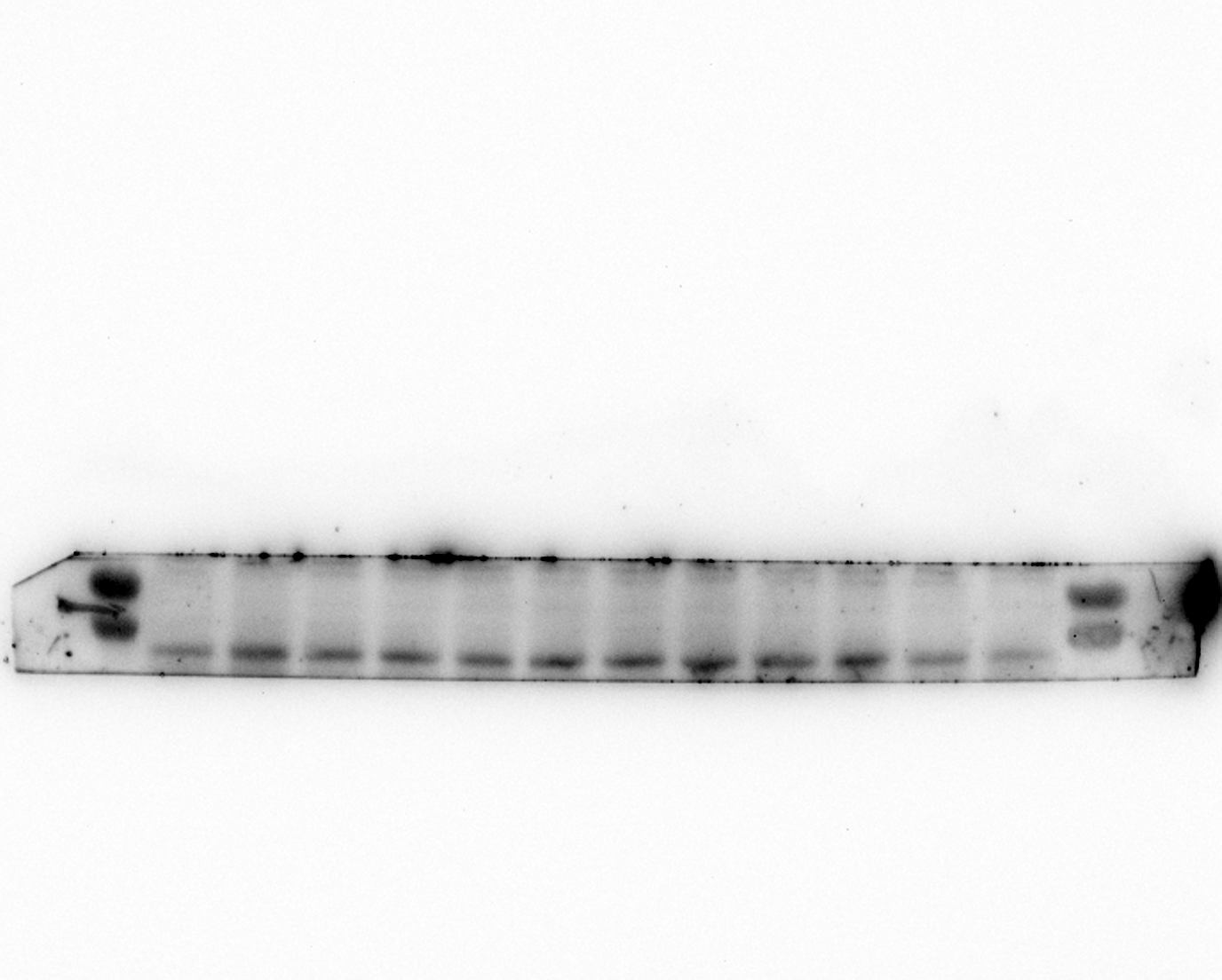

Supplement: Supplementary file 5 — Source Data for Figure 4 [file EMBJ-39-e103304-s003.zip › Fig_4T-Tubulin_(n=2)(Lane1-8)(2).Tif]

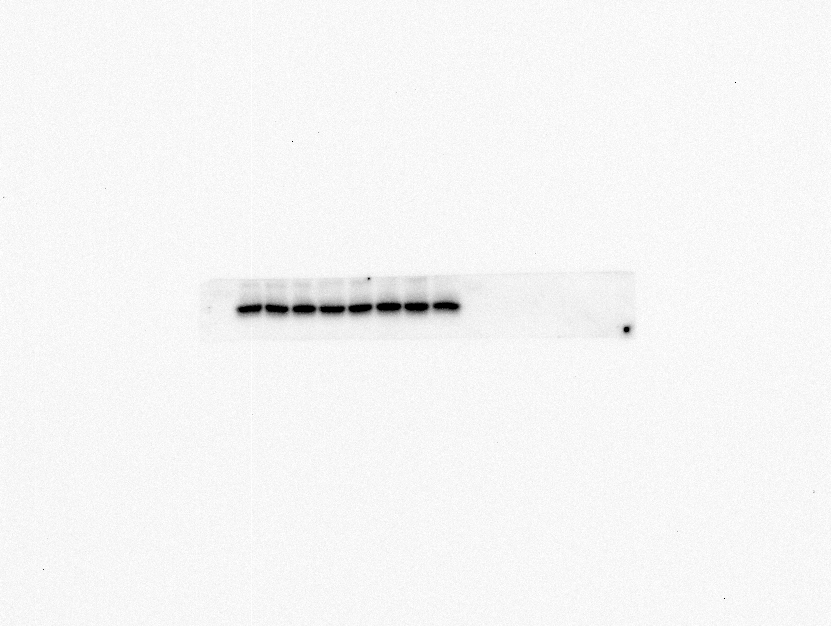

Supplement: Supplementary file 5 — Source Data for Figure 4 [file EMBJ-39-e103304-s003.zip › Fig_4T-Tubulin_(n=2)(Lane1-8)(representative_picture).png]

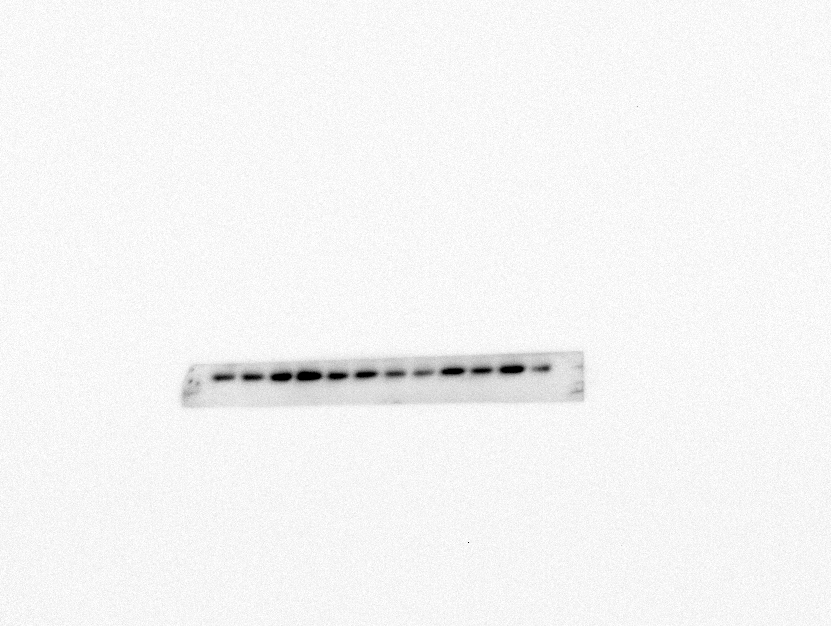

Supplement: Supplementary file 5 — Source Data for Figure 4 [file EMBJ-39-e103304-s003.zip › Fig_4T-UCP1_(n=2)(Lane1-8)(2).png]

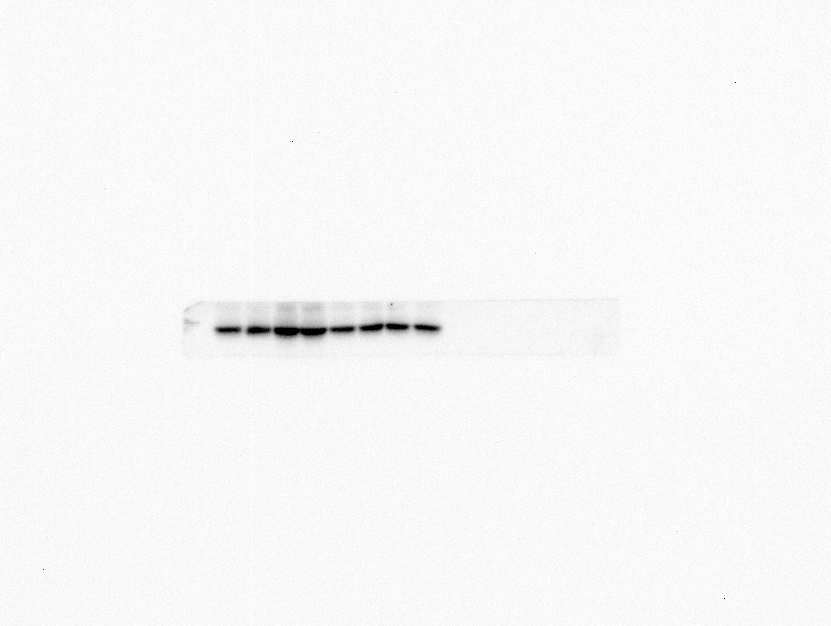

Supplement: Supplementary file 5 — Source Data for Figure 4 [file EMBJ-39-e103304-s003.zip › Fig_4T-UCP1_(n=2)(Lane1-8)(representative_picture).png]

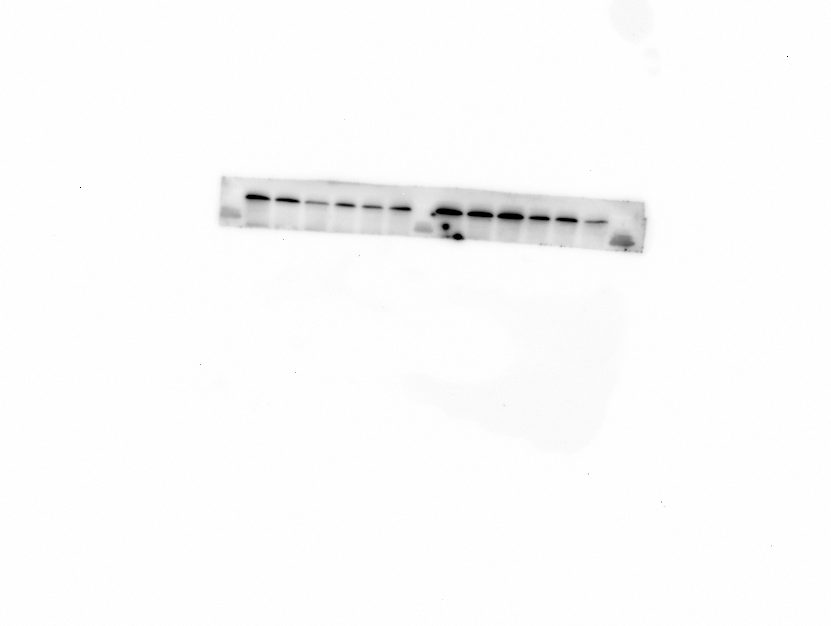

Supplement: Supplementary file 6 — Source Data for Figure 5 [file EMBJ-39-e103304-s004.zip › Fig_5E-OXGR1_(n=3)(Lane7-12)(representative_picture).png]

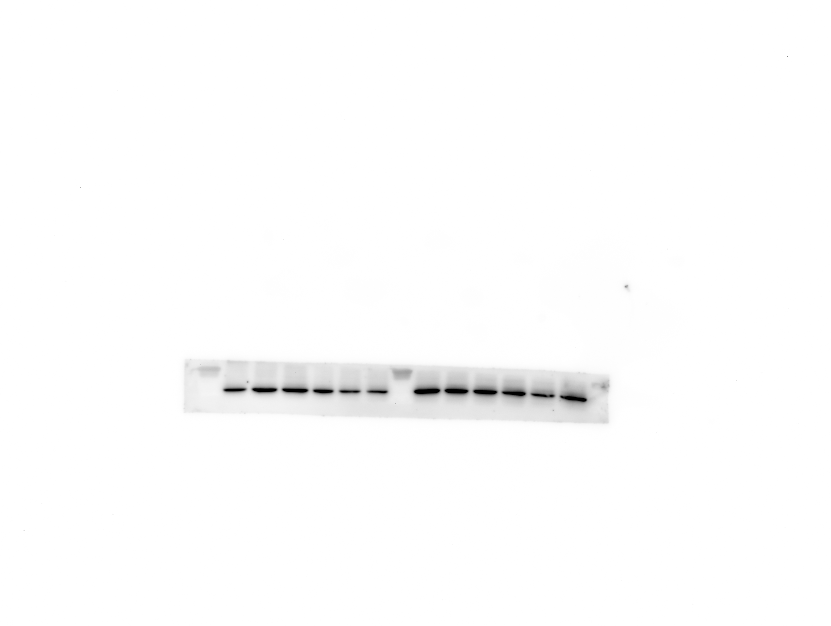

Supplement: Supplementary file 6 — Source Data for Figure 5 [file EMBJ-39-e103304-s004.zip › Fig_5E-Tubulin_(n=3)(Lane7-12)(representative_picture).png]

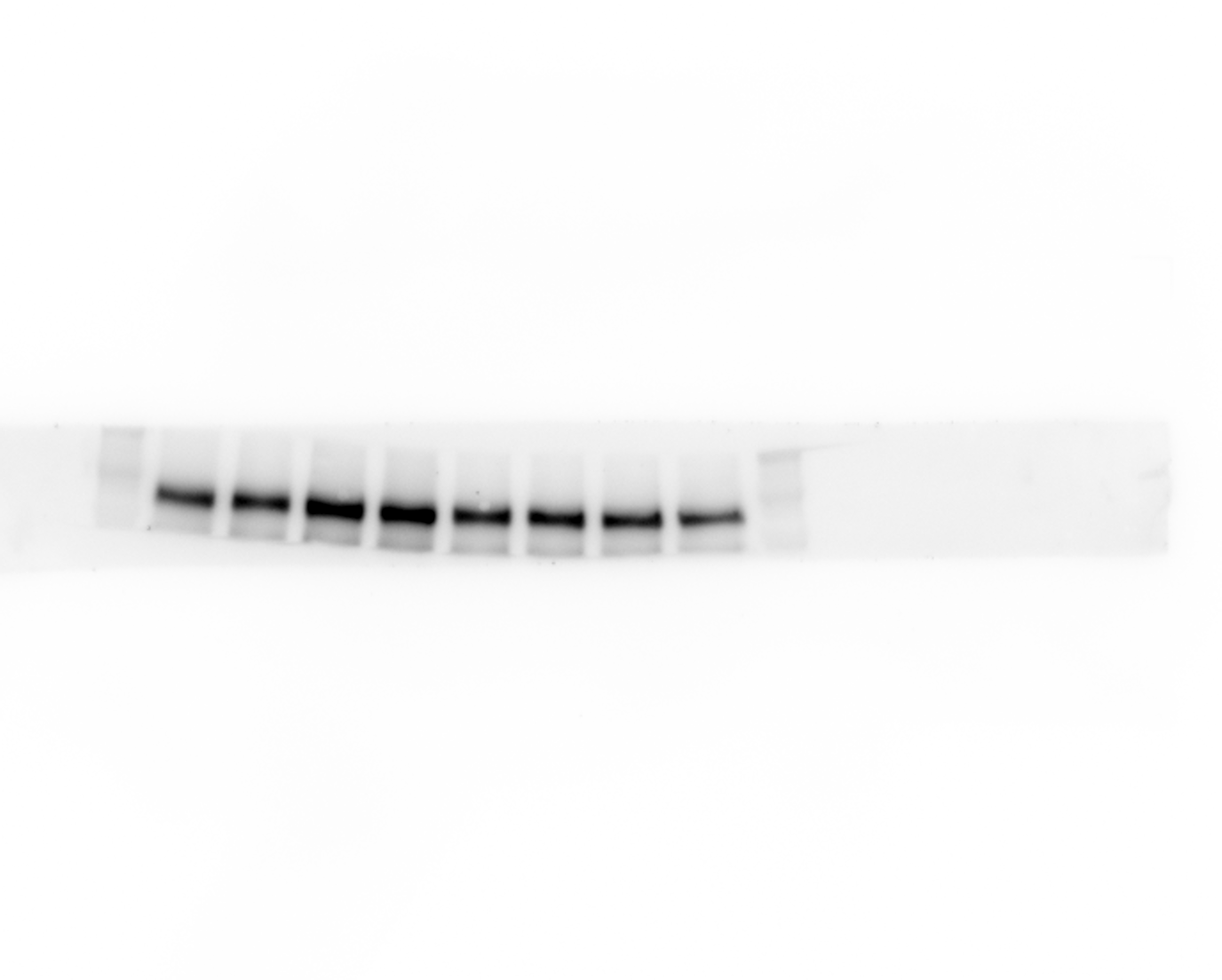

Supplement: Supplementary file 6 — Source Data for Figure 5 [file EMBJ-39-e103304-s004.zip › Fig_5N-ATGL_(n=2)(Lane1-8)(2).Tif]

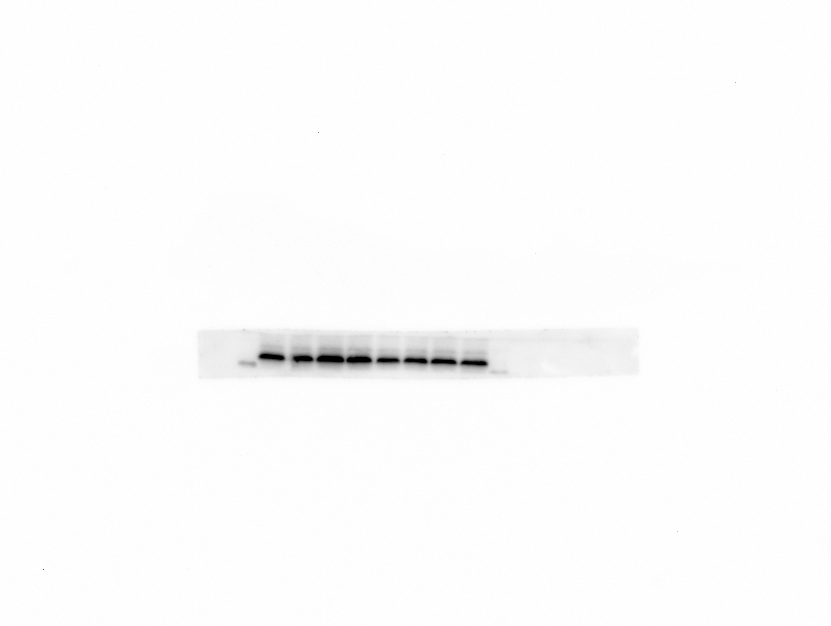

Supplement: Supplementary file 6 — Source Data for Figure 5 [file EMBJ-39-e103304-s004.zip › Fig_5N-ATGL_(n=2)(Lane1-8)(representative_picture).png]

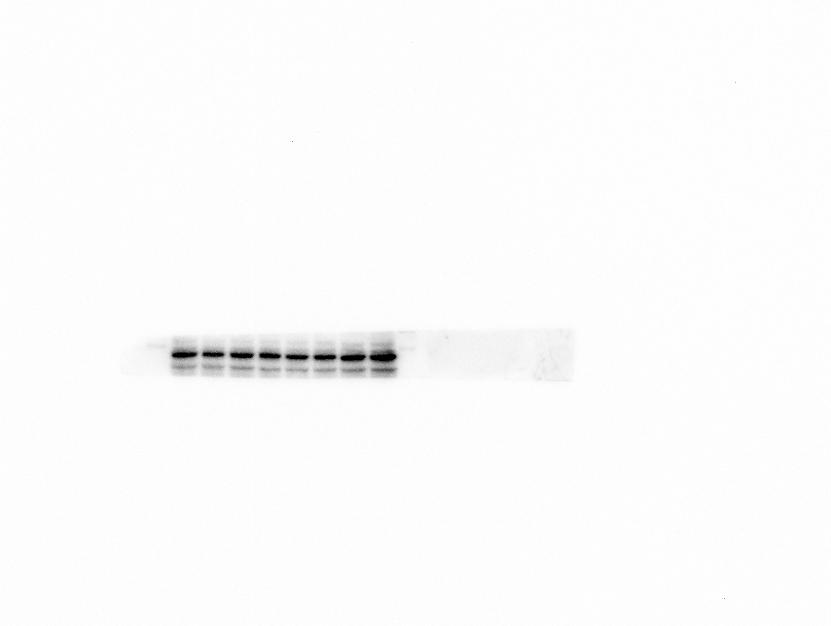

Supplement: Supplementary file 6 — Source Data for Figure 5 [file EMBJ-39-e103304-s004.zip › Fig_5N-HSL_(n=2)(Lane1-8)(2).png]

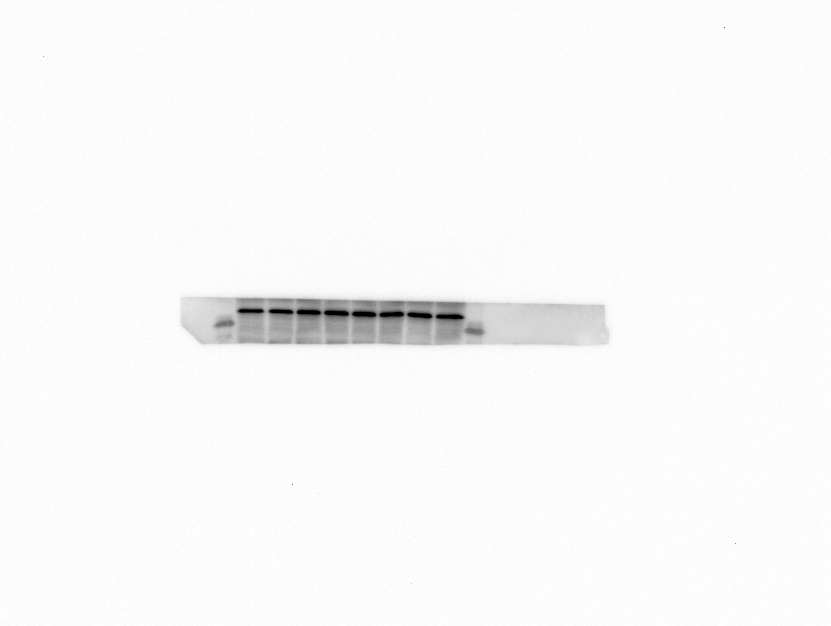

Supplement: Supplementary file 6 — Source Data for Figure 5 [file EMBJ-39-e103304-s004.zip › Fig_5N-HSL_(n=2)(Lane1-8)(representative_picture).png]

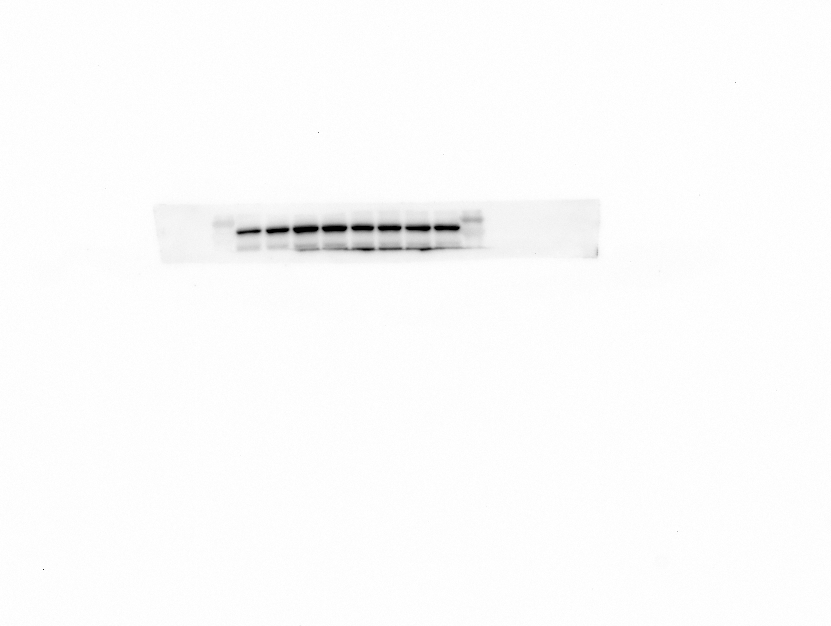

Supplement: Supplementary file 6 — Source Data for Figure 5 [file EMBJ-39-e103304-s004.zip › Fig_5N-p-HSL_(n=2)(Lane1-8)(2).png]

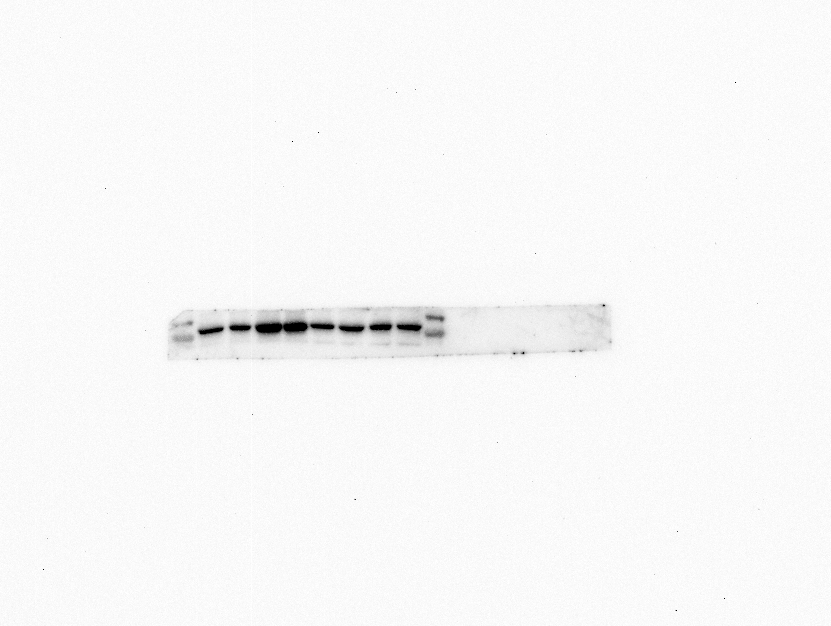

Supplement: Supplementary file 6 — Source Data for Figure 5 [file EMBJ-39-e103304-s004.zip › Fig_5N-p-HSL_(n=2)(Lane1-8)(representative_picture).png]

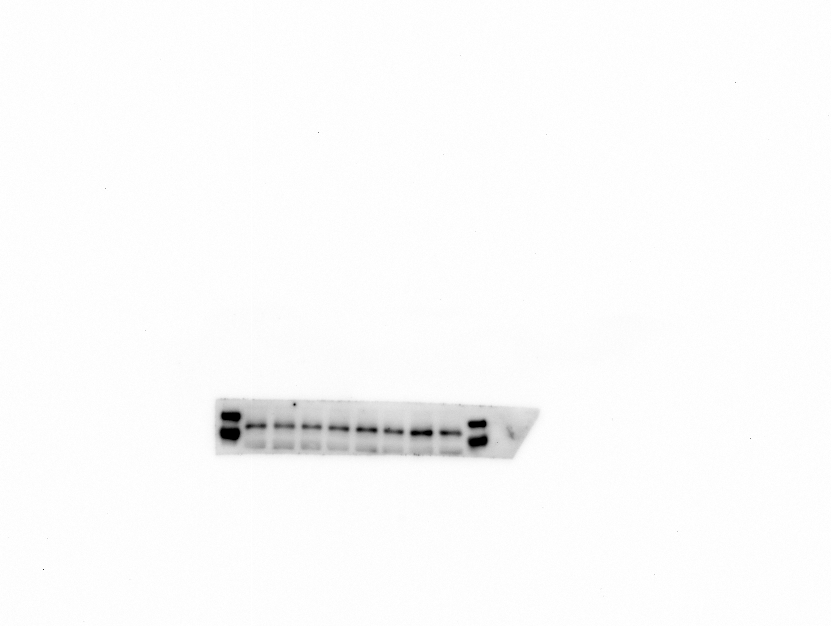

Supplement: Supplementary file 6 — Source Data for Figure 5 [file EMBJ-39-e103304-s004.zip › Fig_5N-Tubulin_(n=2)(Lane1-8)(2).png]

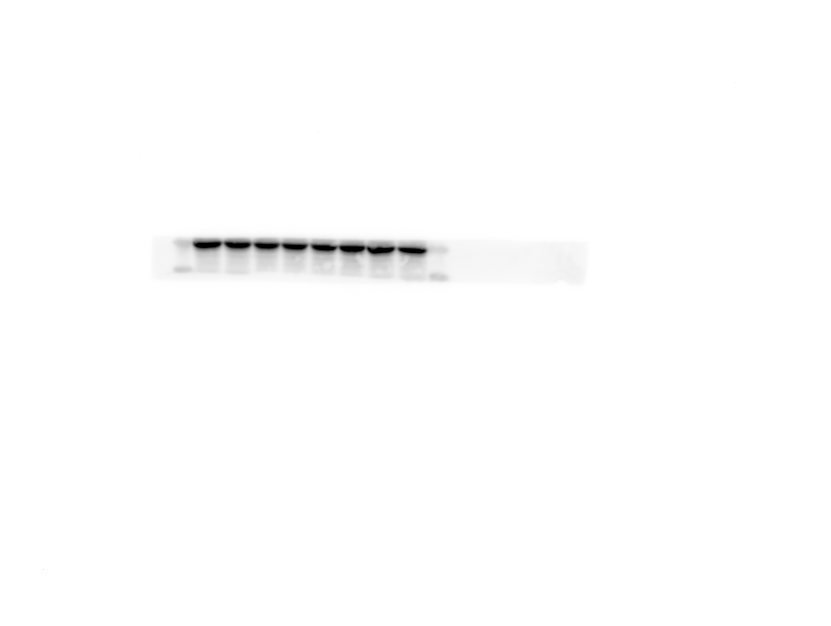

Supplement: Supplementary file 6 — Source Data for Figure 5 [file EMBJ-39-e103304-s004.zip › Fig_5N-Tubulin_(n=2)(Lane1-8)(representative_picture).png]
